# Effect of tailoring biliopancreatic limb length based on total small bowel length *versus* standard limb length in one anastomosis gastric bypass: 1-year outcomes of the TAILOR randomized clinical superiority trial

**DOI:** 10.1093/bjs/znae219

**Published:** 2024-08-30

**Authors:** Nienke Slagter, Lindsy van der Laan, Loek J M de Heide, Ewoud H Jutte, Mirjam A Kaijser, Stefan L Damen, André P van Beek, Marloes Emous

**Affiliations:** Center for Obesity Northern Netherlands, Department of Bariatric and Metabolic Surgery, Medical Center Leeuwarden, Leeuwarden, The Netherlands; Department of Surgery, Medical Center Leeuwarden, Leeuwarden, The Netherlands; Postgraduate School of Medicine, University of Groningen, University Medical Center Groningen, Groningen, The Netherlands; Department of Endocrinology, University of Groningen, University Medical Center Groningen, Groningen, The Netherlands; Center for Obesity Northern Netherlands, Department of Bariatric and Metabolic Surgery, Medical Center Leeuwarden, Leeuwarden, The Netherlands; Department of Surgery, Medical Center Leeuwarden, Leeuwarden, The Netherlands; Postgraduate School of Medicine, University of Groningen, University Medical Center Groningen, Groningen, The Netherlands; Department of Endocrinology, University of Groningen, University Medical Center Groningen, Groningen, The Netherlands; Center for Obesity Northern Netherlands, Department of Bariatric and Metabolic Surgery, Medical Center Leeuwarden, Leeuwarden, The Netherlands; Department of Surgery, Medical Center Leeuwarden, Leeuwarden, The Netherlands; Center for Obesity Northern Netherlands, Department of Bariatric and Metabolic Surgery, Medical Center Leeuwarden, Leeuwarden, The Netherlands; Department of Surgery, Medical Center Leeuwarden, Leeuwarden, The Netherlands; Center for Obesity Northern Netherlands, Department of Bariatric and Metabolic Surgery, Medical Center Leeuwarden, Leeuwarden, The Netherlands; Department of Surgery, Medical Center Leeuwarden, Leeuwarden, The Netherlands; Center for Obesity Northern Netherlands, Department of Bariatric and Metabolic Surgery, Medical Center Leeuwarden, Leeuwarden, The Netherlands; Department of Surgery, Medical Center Leeuwarden, Leeuwarden, The Netherlands; Postgraduate School of Medicine, University of Groningen, University Medical Center Groningen, Groningen, The Netherlands; Department of Endocrinology, University of Groningen, University Medical Center Groningen, Groningen, The Netherlands; Center for Obesity Northern Netherlands, Department of Bariatric and Metabolic Surgery, Medical Center Leeuwarden, Leeuwarden, The Netherlands; Department of Surgery, Medical Center Leeuwarden, Leeuwarden, The Netherlands

## Abstract

**Background:**

Tailoring the biliopancreatic limb length in one anastomosis gastric bypass is proposed as beneficial in retrospective studies, yet randomized trials are lacking. The aim of this double-blind, single-centre RCT was to ascertain whether tailoring biliopancreatic limb length based on total small bowel length (TSBL) results in superior outcomes after one anastomosis gastric bypass compared with a fixed 150 cm biliopancreatic limb length.

**Methods:**

Eligible patients, meeting International Federation for the Surgery of Obesity and Metabolic Disorders (IFSO) criteria for metabolic bariatric surgery, scheduled for primary one anastomosis gastric bypass surgery, and willing to be randomized, underwent TSBL measurement during surgery. When TSBL measurement was feasible, patients were randomly assigned to a standard 150 cm biliopancreatic limb length or a tailored biliopancreatic limb based on TSBL: TSBL less than 500 cm, biliopancreatic limb 150 cm; TSBL 500–700 cm, biliopancreatic limb 180 cm; and TSBL greater than 700 cm, biliopancreatic limb 210 cm. The primary outcome was percentage total weight loss at 5 years.

**Results:**

Between September 2020 and August 2022, 212 patients were randomized into the standard biliopancreatic limb group (105 patients) or the tailored biliopancreatic limb group (107 patients). The mean(s.d.) TSBL was 657(128) cm (range 295–1020 cm). In the tailored group, 150, 180, and 210 cm biliopancreatic limb lengths were applied to 8.4%, 53.3%, and 38.3% of patients respectively. The mean(s.d.) 1-year percentage total weight loss was 32.8(6.9)% in the standard group and 33.1(6.2)% in the tailored group (*P* = 0.787). Nutritional deficiencies and short-term complications showed no significant differences.

**Conclusion:**

Tailoring biliopancreatic limb length based on TSBL is safe and feasible. One year after surgery, it is not superior to a standard biliopancreatic limb length of 150 cm in terms of percentage total weight loss.

**Registration number:**

Dutch Trial Register, NL7945.

## Teaser

The 1-year follow-up results of an RCT are presented, demonstrating that tailoring the biliopancreatic limb length based on total small bowel length is a safe and feasible technique. One year after surgery, this tailored approach does not exhibit superiority over a fixed biliopancreatic limb length in terms of percentage total weight loss.

## Introduction

Obesity is a paramount public health concern^1^. Metabolic bariatric surgery (MBS) is the most effective and sustainable treatment for obesity, resulting in good weight loss, resolution of associated co-morbidities, and improved life expectancy and quality of life at long-term follow-up^2–5^. In 2018, the one anastomosis gastric bypass (OAGB) was recognized as an endorsed MBS procedure by the International Federation for the Surgery of Obesity and Metabolic Disorders (IFSO). Currently, it is the third most commonly performed MBS procedure worldwide, with its popularity expanding globally^6,7^. The OAGB consists of a single gastrojejunal anastomosis connecting a long gastric pouch with a jejunal omega loop. It is a technically less demanding procedure compared with the ‘gold standard’ of Roux-en-Y gastric bypass (RYGB). According to the existing literature, OAGB seems to provide at least similar weight loss results compared with RYGB, but with potentially more nutritional issues, diarrhoea, and gastro-oesophageal reflux disease (GERD) symptoms^8–13^.

In recent years, a longer biliopancreatic (BP) limb has been found to be an important factor in establishing sufficient weight loss^14–18^. In contrast, an extended BP limb can result in problems, such as vitamin deficiencies, protein malnutrition, and diarrhoea^14,15,18–20^. Therefore, an appropriate BP limb length aims to achieve maximum weight loss, while minimizing adverse effects, such as nutritional deficiencies and gastrointestinal symptoms. Similar to RYGB, there are currently no established guidelines for determining the optimal BP limb length in OAGB. The determination of the BP limb length exhibits considerable variation among bariatric surgeons, as some use a fixed length ranging from 150 to more than 250 cm, whereas others tailor the length based on factors such as BMI, age, sex, or co-morbidities^8,15,21–23^. The available literature on BP limb length in OAGB consists of retrospective studies and the presence of subjective variations limits the comparability of these studies.

The effect of a certain BP limb length on outcomes may be affected by the considerable individual variability of total small bowel length (TSBL). In the most recent IFSO statement on OAGB, a majority of surgeons recommended using 30–40% of the TSBL as the BP limb length^24^. This recommendation is based on the wide variation in TSBL among individuals, as reported TSBL ranges from 350 to over 1000 cm^25^. To further explore the IFSO statement proposal, the aim of the TAILOR RCT was to assess whether tailoring the BP limb based on TSBL is superior to a standard fixed 150 cm BP limb in terms of percentage total weight loss (%TWL) at 5 years and the specific aim of this predefined 1-year analysis of the RCT was to report the initial %TWL and safety outcomes.

## Methods

### Study design

The TAILOR study was a double-blind, randomized clinical superiority trial conducted in a non-academic teaching hospital located in the north of the Netherlands with a 5-year follow-up. The medical ethics committee (Regionale Toetsingscommissie Patiëntgebonden Onderzoek, Leeuwarden, NR 1082) approved the study and the study was prospectively registered on 8 September 2019 (Dutch Trial Register, NL7945). The trial was performed in accordance with the Declaration of Helsinki. All patients provided written informed consent.

The complete study protocol was published online^26^ and is available as *File S1*. All patients complied with the IFSO guidelines for MBS and underwent screening by a multidisciplinary team. To be eligible, patients had to be aged between 18 and 65 years and scheduled for primary OAGB surgery with a BMI of 40 kg/m^2^ or higher, or 35 kg/m^2^ with the presence of at least one co-morbidity. Intraoperatively complete TSBL had to be measurable intraoperatively. Exclusion criteria included: BMI greater than 50 kg/m^2^; preoperative deficiencies of vitamin B_12_, D, or iron (ferritin); use of extra (multi-) vitamin supplements except for vitamin D (max 800 IU/day); intolerance of multivitamin FitForMe (FFM) weight loss surgery (WLS) Primo; pregnancy planning within the first 2 years after surgery; history of gastrointestinal disease or abdominal surgery that could prevent the measurement of TSBL; addiction behaviour; and renal or hepatic insufficiency.

### Intervention, randomization, and blinding

After successful measurement of the TSBL by the surgeon, participants were peroperatively randomly assigned to either the standard BP limb group or the intervention tailored BP limb group. In the standard group, a BP limb length of 150 cm was used, whereas, in the intervention group, the BP limb length was based on TSBL using the following three predefined categories: first, for a TSBL shorter than 500 cm, a BP limb length of 150 cm was used; second, for a TSBL ranging between 500 and 700 cm, a BP limb length of 180 cm was used; and third, for a TSBL longer than 700 cm, a BP limb length of 210 cm was used. The randomization process was performed via a sealed envelope method during the operation using a computer-generated block randomization with a block size of 30 and 1 : 1 allocation administered by an independent party. Only the operating surgeon and the operating room team were aware of the allocation. The treatment allocation was documented in a secured coded database by an employee outside the research team. The patients, the research team, and other care providers of the MBS department were all blinded to the treatment allocation.

### Study procedure

The standard preoperative workup comprised several months of counselling by a dietician to prepare individuals for the postoperative lifestyle regimen. Additionally, a multidisciplinary team conducted a thorough screening, assessing mental health, detailed medical conditions, and adherence to the postoperative lifestyle. Before surgery, patients adhered to a diet rich in protein and low in sugar and fat for a duration of 2 or 3 weeks, depending on their BMI. The surgical technique of the OAGB procedure complied with the technique outlined by the IFSO^6^. The gastric pouch was created using a 34-Fr calibration tube, which was held toward the lesser curvature. After the creation of the gastric pouch, the TSBL was measured. The small bowel was measured from the ligament of Treitz using the stepwise hand-over-hand technique, using two marked graspers to pass the small bowel in estimated steps of 5 cm^27^. The length of the antimesenteric border was measured with the bowel in a normal position and without applying traction. After the creation of the gastrojejunostomy, an anti-reflux suture was placed, using one absorbable suture, as described previously^28^. Petersen’s space was closed. All OAGB procedures were performed by four experienced metabolic bariatric surgeons.

After MBS, a lifelong daily intake of multivitamins is recommended and they should be tailored to MBS. To standardize vitamin supplementation throughout the study, patients were provided with daily multivitamin FFM WLS Primo during the 5-year follow-up. All patients were also prescribed 800 IU vitamin D and 1000 mg calcium per day, as part of standard care.

Follow-up appointments were conducted according to usual care, consisting of visits at 6 and 18 months, and yearly visits for up to 5 years. During each follow-up visit, standard care included: inquiry into the patient’s general well-being and complaints, daily bowel movements, and current medication usage; assessment of co-morbidities; and measurement of body weight, blood pressure, and pulse. To ensure accuracy and consistency, for all participants, their exact follow-up visits were determined, and patients were scheduled within a maximum window of 2 weeks before or 1 week after this specified date to minimize variability in follow-up visits. Blood samples were collected according to the standard protocol of follow-up after bariatric surgery, with an additional 20 ml of blood drawn at 1-, 3-, and 5-year visits to be stored in a freezer for future analysis. The sample analyses were carried out by Certe (Groningen, The Netherlands). Haemoglobin (Hb) levels were determined utilizing routine haematology analysers (XN-Series, Sysmex, Kobe, Japan). Potassium concentrations were measured employing ion-selective electrodes (ISE) on a Roche Cobas 8000 chemistry analyser (Roche, Mannheim, Germany). The bromocresol purple method (Roche, Mannheim, Germany) was employed for assessing albumin levels. Vitamin A, B1, and B6 analyses were performed at University Medical Center Groningen, utilizing a liquid chromatography–tandem mass spectrometry method for quantification. Other laboratory measurements, including ferritin, magnesium, phosphate, calcium, vitamin B12, and vitamin D, were assessed through commercially available assays on the Roche Cobas 8000 chemistry analyser. Zinc levels were determined using a colorimetric method without deproteinization (Instruchemie) on the same Roche Cobas 8000 chemistry analyser. Any vitamin or micronutrient deficiencies were addressed with substitution therapy, as per standard care.

### Outcome measures

The primary outcome of this RCT was %TWL at 5-year follow-up. The outcomes to be addressed in this predefined 1-year analysis were %TWL and safety. %TWL was defined as (initial weight–postoperative weight)/initial weight × 100. Baseline weight was recorded on the day of the surgery. The secondary outcomes, assessed in both the predefined 1-year analysis and the 5-year follow-up, included: percentage excess weight loss (%EWL); the proportion of patients with a BMI between 22 and 30 kg/m^2^; daily bowel movements; early and late complications; remission of type 2 diabetes mellitus (T2DM), hypertension, and obstructive sleep apnoea (OSA); nutritional deficiencies; quality of life, assessed using the questionnaire for patients with obesity (OBESI-Q) (version 1)^29,30^; and the percentage of patients experiencing dumping symptoms, determined using the Dumping Severity Score (DSS)^31^. %EWL was defined as (initial weight–postoperative weight)/(initial weight–ideal weight) × 100, with ideal weight defined as the weight corresponding to a BMI of 25 kg/m^2^. Total remission of T2DM was defined as an HbA1c of less than 48 mmol/mol without diabetes medication at the time of the follow-up visit^32^. Improvement of diabetes was defined as a reduction in HbA1c of 10 mmol/mol or more compared with the previous follow-up visit, but not reaching the remission criterion, and/or a lower number and/or a lower dosage of anti-diabetic medications. The definition of total remission of hypertension was being able to discontinue all antihypertensive medication, whereas improvement of hypertension was defined as a reduction in antihypertensive medication. Resolution of OSA was defined as the cessation of continuous positive airway pressure (CPAP) or other devices used.

Nutritional deficiencies were defined using the reference values of the local laboratory (Certe, Groningen, The Netherlands) and are shown in *Table S1*^33^. The authors’ bariatric centre utilized the authors’ own defined cut-off values for ferritin, vitamin B12, and vitamin A to initiate supplementation. Iron deficiency was defined as a ferritin level below 30 µg/l or 30–50 µg/l and a decrease of over 50 µg/l compared with the prior measurement. Vitamin B12 and vitamin A deficiency were defined as levels below 250 pmol/l and 0.8 µmol/l respectively.

For dumping symptom assessment, the DSS questionnaire was used. The questionnaire asks patients to rate the intensity of eight early dumping symptoms and six late dumping symptoms using a four-point Likert scale; early dumping was identified when patients experienced three or more early dumping symptoms with intensity scores of two or three (that is moderate or severe), including at least one autonomic symptom, and late dumping was identified when patients experienced three or more late dumping symptoms with intensity scores of two or three (that is moderate or severe), including at least one neuroglycopenic symptom^31^. During each follow-up visit, the presence of GERD was noted and assessed using the GerdQ tool. The GerdQ tool comprises six items, four positive and two negative predictors of GERD, and can be used to diagnose GERD^34^. GERD was defined as symptoms of reflux with a GerdQ score greater than or equal to eight or complaints of reflux reported between follow-up appointments.

### Statistical analysis and sample size

The study hypothesized that the mean %TWL in the tailored BP limb group would be 5% more than that in the standard group (40% and 35% respectively). The mean 5% difference was chosen to uncover differences that hold clinical significance, as attaining an extra weight loss change of greater than or equal to 5% of initial body weight had been associated with improved overall quality of life and remission or improvement of co-morbidities^35^. To achieve 80% power to identify a 5% difference with estimated group standard deviations of 12 and an α level of 0.05, a sample size of 92 patients per group was required. With an expected maximum dropout rate of 15% due to conversion of OAGB to RYGB, the calculated sample size was 106 patients in each group, leading to a total of 212 participants enrolled in the study^28^.

**Fig. 1:**
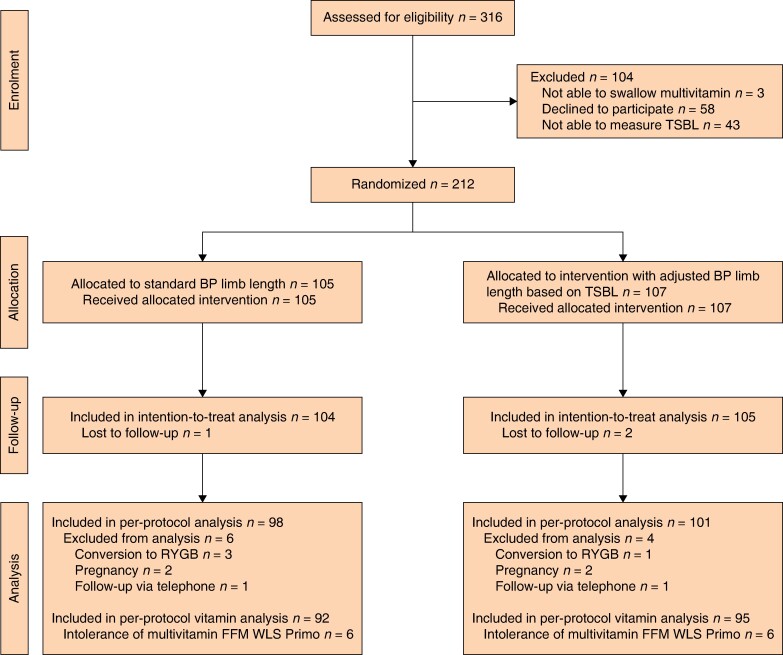
Flow diagram of participants in the TAILOR study CONSORT flow diagram for randomized trials. TSBL, total small bowel length; BP, biliopancreatic; RYGB, Roux-en-Y gastric bypass; FFM, FitForMe; WLS, weight loss surgery.

The study examined primary and secondary outcomes by analysing both intention-to-treat and per-protocol populations (*Fig. 1*). The per-protocol group was refined by excluding patients displaying significant deviations from the prescribed protocol, including pregnancy, conversion to RYGB, and follow-up via telephone. In terms of nutritional analyses, participants who used alternative multivitamins, instead of the multivitamin FFM WLS Primo, were classified as instances of protocol deviation, subsequently leading to their exclusion from the per-protocol assessments. According to the study protocol, the per-protocol analysis set was invoked only when the proportion of excluded patients attained a noteworthy threshold exceeding 15% of the comprehensive analysis set. Given the limited occurrence of protocol deviations and the similarity in results when compared with the intention-to-treat population, only the intention-to-treat population’s findings are presented.

Normally distributed continuous variables are presented as mean(s.d.) and were analysed using a two-sample *t* test. Categorical outcomes were assessed using a chi-squared test or Fisher’s exact test. Changes in quality-of-life scores compared with baseline were assessed using a paired *t* test. *P* ≤ 0.050 was considered statistically significant. The statistical analysis was conducted using SPSS^®^ (IBM, Armonk, NY, USA; version 28).

## Results

A total of 316 patients scheduled to undergo primary OAGB between July 2020 and September 2022 were initially assessed for eligibility (*Fig. 1*). Out of these patients, 3 patients (0.9%) were not able to swallow the multivitamin FFM WLS Primo, 58 patients (18.4%) declined to participate, and TSBL could not be measured intraoperatively in 43 patients (13.6%) (due to adhesions in 42 patients (97.7%) and hepatomegaly in 1 patient (2.3%)). Ultimately, a total of 212 patients were randomly assigned to either a standard BP limb length (105 patients) or a tailored BP limb length based on TSBL (107 patients). At 1 year, three patients were lost to follow-up. The baseline characteristics of the patients are shown in *Table 1*. The mean(s.d.) age was 44.1(10.9) years and 187 patients (88.2%) were female. The mean(s.d.) preoperative BMI was 40.8(3.7) kg/m^2^ and the mean(s.d.) operating time was 49.8(10.9) min. The mean(s.d.) TSBL was 644.8(124.8) cm in the group with the standard BP limb length and 669.5(130.6) cm in the group with the tailored BP limb length. In the tailored group, three different BP limb lengths were applied based on TSBL: 150 cm in 9 patients (8.4%), 180 cm in 57 patients (53.3%), and 210 cm in 41 patients (38.3%). The mean(s.d.) TSBL measurement time was 6:28(3:18) min (204 patients). In one patient, a small bowel perforation occurred, possibly associated with measuring TSBL, though it was unclear whether the perforation happened during the measurement of the BP limb or TSBL. Furthermore, there were no complications associated with TSBL measurement.

**Table 1:** Baseline characteristics

	Standard BP limb length, *n* = 105	Tailored BP limb length based on TSBL, *n* = 107
**Sex**		
Male	11 (10.5)	14 (13.1)
Female	94 (89.5)	93 (86.9)
Age (years), mean(s.d.)	43.0 (10.9)	45.1 (10.8)
Preoperative weight (kg), mean(s.d.)	120.9 (16.8)	117.0 (14.2)
Height (cm), mean(s.d.)	171.4 (8.0)	169.8 (7.9)
BMI (kg/m^2^), mean(s.d.)	41.1 (3.7)	40.5 (3.6)
Hypertension	23 (21.9)	40 (37.4)
Type 2 diabetes mellitus	15 (14.3)	14 (13.1)
Cardiovascular disease	2 (1.9)	8 (7.5)
Sleep apnea	16 (15.2)	18 (16.8)
Cholecystectomy	12 (11.4)	16 (15.0)
Operating time (min), mean(s.d.)	50.4 (11.3)	49.2 (10.5)
TSBL (cm), mean(s.d.) (min–max)	644.8 (124.8) (355–1020)	669.5 (130.6) (295–990)
**BP limb length (cm)**		
150	105 (100.0)	9 (8.4)
180	0 (0.0)	57 (53.3)
210	0 (0.0)	41 (38.3)
Common limb length (cm), mean(s.d.) (min–max)	494.8 (124.8) (205–870)	480.5 (115.1) (145–780)

Values are *n* (%) unless otherwise indicated. BP, biliopancreatic; TSBL, total small bowel length.

**Fig. 2:**
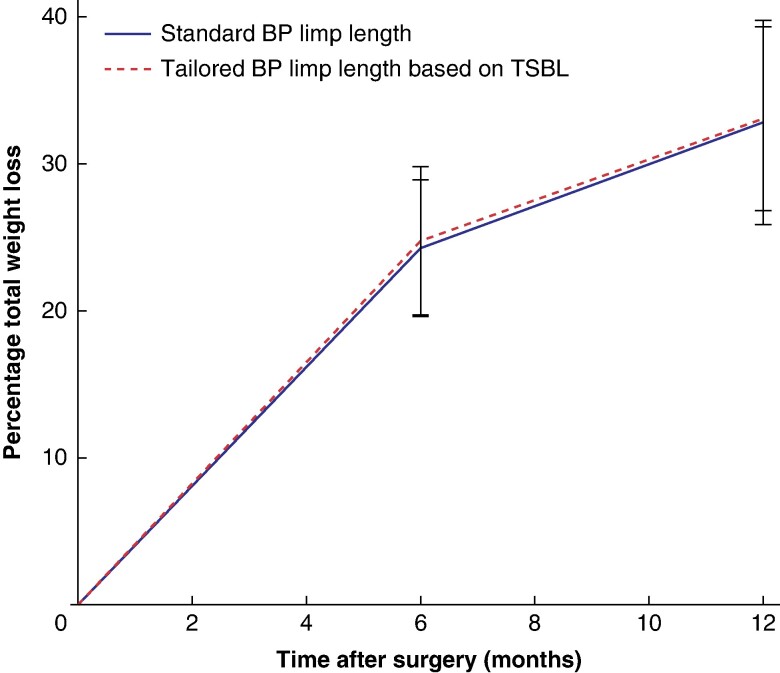
Percentage total weight loss during the first year after surgery Values are mean(s.d.). BP, biliopancreatic; TSBL, total small bowel length.

After a 1-year follow-up, no statistically significant differences were observed in %TWL, BMI, and %EWL between the two groups (*Table 2* and *Fig. 2*). Nutritional deficiencies were evaluated at both 6 and 12 months after surgery. *Table 3* provides an overview of all the nutritional deficiencies observed in the first year and the absolute values of nutritional status at 1-year follow-up. No statistically significant differences were observed between the two groups regarding the frequencies of nutritional deficiencies. Vitamin D deficiency was found in 6.7% of patients in the group with the standard BP limb length and in 14.3% of patients in the group with tailored BP limb length based on TSBL (*P* = 0.075). Iron deficiency was observed in 8.7% of patients in the standard group and 10.5% of patients in the tailored group (*P* = 0.654). There was also no significant difference between the two groups regarding the frequency of vitamin B12 deficiency, which was 4.8% in the standard group and 10.5% in the tailored group (*P* = 0.123). Hypoalbuminaemia (<35 g/l) was seen in 29.8% of patients in the standard group and 30.5% of patients in the tailored group (*P* = 0.916). The percentage of patients with normal vitamin D, B12, and iron at 1 year without extra vitamin supplementation was 82.7% in the group with the standard BP limb length and 73.1% in the group with tailored BP limb length based on TSBL (*P* = 0.095).

**Table 2:** Weight loss outcomes 1 year after surgery for the intention-to-treat population

	Standard BP limb length, *n* = 104 (missing data *n* = 1)	Tailored BP limb length based on TSBL, *n* = 105 (missing data *n* = 2)	*P*
BMI (kg/m^2^)	27.5 (3.7)	27.1 (3.4)	0.392
Δ BMI (kg/m^2^)	13.5 (3.2)	13.4 (2.9)	0.876
%EWL	87.0 (22.5)	89.0 (20.3)	0.505
%TWL	32.8 (6.9)	33.1 (6.2)	0.787
BMI 22–30 kg/m^2^, *n* (%)	73 (70.2)	83 (79.0)	0.155

Values are mean(s.d.) unless otherwise indicated. BP, biliopancreatic; TSBL, total small bowel length; %EWL, percentage excess weight loss; %TWL, percentage total weight loss.

**Table 3:** Nutritional deficiencies and absolute values of nutritional status

	Standard BP limb length, *n* = 104 (missing data *n* = 1)	Tailored BP limb length based on TSBL, *n* = 105 (missing data *n* = 2)	*P*
Nutritional deficiencies in the first year after surgery*			
Anaemia	14 (13.5)	14 (13.3)	0.978
Iron deficiency	9 (8.7)	11 (10.5)	0.654
Hypokalaemia	10 (9.6)	13 (12.4)	0.523
Hypomagnesaemia	1 (1.0)	0 (0.0)	0.498
Hypophosphataemia	14 (13.5)	11 (10.5)	0.506
Hypoalbuminaemia			
<35 g/l	31 (29.8)	32 (30.5)	0.916
<33 g/l	9 (8.7)	9 (8.6)	0.983
Hypocalcaemia	0 (0.0)	2 (1.9)	0.498
Vitamin A deficiency	6 (5.8)	6 (5.7)	0.986
Vitamin B1 deficiency	5 (4.8)	3 (2.9)	0.498
Vitamin B6 deficiency	1 (1.0)	0 (0.0)	0.498
Vitamin B12 deficiency	5 (4.8)	11 (10.5)	0.123
Vitamin D deficiency	7 (6.7)	15 (14.3)	0.075
Folic acid deficiency	5 (4.8)	4 (3.8)	0.748
Zinc deficiency	1 (1.0)	1 (1.0)	>0.999
Normal vitamin D, B12, and iron at 1 year without extra vitamin supplementation	86 (82.7)	76 (73.1)	0.095
**Absolute values of nutritional status at 1-year follow-up**			
Haemoglobin (mmol/l), mean(s.d.)	8.3 (0.7)	8.2 (0.7)	0.759
Ferritin (µg/l), mean(s.d.)	156.7 (103.2)	164.9 (122.4)	0.601
Potassium (mmol/l), mean(s.d.)	4.0 (0.3)	3.9 (0.4)	0.142
Magnesium (mmol/l), mean(s.d.)	0.9 (0.1)	0.9 (0.1)	0.187
Phosphate (mmol/l), mean(s.d.)	1.1 (0.2)	1.1 (0.2)	0.807
Albumin (g/l), mean(s.d.)	36.5 (2.5)	36.5 (3.1)	0.980
Calcium (mmol/l), mean(s.d.)	2.4 (0.1)	2.3 (0.1)	0.018
Vitamin A (µmol/l), mean(s.d.)	1.5 (0.4)	1.5 (0.4)	0.402
Vitamin B1 (nmol/l), mean(s.d.)	167.4 (32.9)	166.8 (35.0)	0.908
Vitamin B6 (nmol/l), mean(s.d.)	96.0 (29.0)	89.6 (24.1)	0.087
Vitamin B12 (pmol/l), mean(s.d.)	637.3 (288.7)	615.8 (285.5)	0.590
Vitamin D (nmol/l), mean(s.d.)	91.1 (26.0)	82.9 (25.9)	0.023
Folic acid (nmol/l), mean(s.d.)	30.2 (12.3)	26.8 (12.6)	0.053
Zinc (µmol/l), mean(s.d.)	13.4 (2.1)	13.4 (2.4)	0.805

Values are *n* (%) unless otherwise indicated. *Nutritional deficiencies were evaluated at both 6 and 12 months after surgery, with the results from these two evaluations combined and presented in the table. BP, biliopancreatic; TSBL, total small bowel length.

*Table 4* shows the serious adverse events associated with surgery with no differences in complication rates between the two groups. Short-term complications, such as bleeding, anastomotic leakage, and intraoperative bowel injury, were observed in 1.9% of the standard group and 3.8% of the tailored group (*P* = 0.683). Problems related to intake/passage in the first year that required enteral or supplementary feeding were seen in 6.7% of the standard group and 1.9% of the tailored group (*P* = 0.101). The frequency of excessive weight loss requiring consultation of a dietician and additional nutrition in the standard and tailored groups was 1.9% and 1.0% respectively (*P* = 0.621). Reoperation was performed in 5.8% of the standard group and 7.6% of the tailored group, with reasons including conversion to RYGB, laparoscopic cholecystectomy for cholelithiasis, cholecystitis, or biliary pancreatitis, as well as bleeding, umbilical hernia, or perforation due to ulcers. Conversion to RYGB within the first year was performed in 1.9% of the patients, with no difference between the two groups (*P* = 0.369). The reasons for conversion to RYGB included biliary reflux (2 patients), stenosis (1 patient), and severe ulcers (1 patient).

**Table 4:** Serious adverse events at 1-year follow-up

	Standard BP limb length, *n* = 104 (missing data *n* = 1)	Tailored BP limb length based on TSBL, *n* = 105 (missing data *n* = 2)	*P*
**Early (≤30 days)**			
Anastomotic leakage	1 (1.0)	0 (0.0)	0.498
Intraoperative bowel injury	0 (0.0)	1 (1.0)	>0.999
Bleeding	1 (1.0)	3 (2.9)	0.621
Nausea and vomiting	0 (0.0)	1 (1.0)	>0.99
Intake/passage problems requiring enteral or supplementary feeding	4 (3.8)	1 (1.0)	0.212
Abdominal pain requiring emergency department visit or admission to hospital	0 (0.0)	2 (1.9)	0.498
**Longer term (>30 days)**			
Nausea and vomiting	1 (1.0)	0 (0.0)	0.498
Intake/passage problems requiring enteral or supplementary feeding	3 (2.9)	1 (1.0)	0.369
Abdominal pain requiring emergency department visit or admission to hospital	5 (4.8)	4 (3.8)	0.748
Excessive weight loss requiring consultation of a dietician and additional nutrition	2 (1.9)	1 (1.0)	0.621
Ulcer	2 (1.9)	1 (1.0)	0.621
Umbilical hernia	0 (0.0)	1 (1.0)	>0.99
Cholecystitis	0 (0.0)	2 (1.9)	0.498
Cholelithiasis	1 (1.0)	0 (0.0)	0.498
Biliary pancreatitis	1 (1.0)	0 (0.0)	0.498
Conversion to RYGB	3 (2.9)	1 (1.0)	0.369
Biliary reflux, *n*	1	1	
Stenosis, *n*	1	0	
Ulcer, *n*	1	0	
Clavien–Dindo grade*			
IIIa	3 (2.9)	0 (0.0)	0.121
IIIb	6 (5.8)	8 (7.6)	0.593
IV/V	0 (0.0)	0 (0.0)	–

Values are *n* (%) unless otherwise indicated. *Clavien–Dindo classification: III, requiring surgical, endoscopic, or radiological intervention; IIIa, intervention under regional/local anaesthesia; IIIb, intervention under general anaesthesia; IV, life-threatening complication requiring intensive care management; and V, patient demise. BP, biliopancreatic; TSBL, total small bowel length; RYGB, Roux-en-Y gastric bypass.

**Fig. 3:**
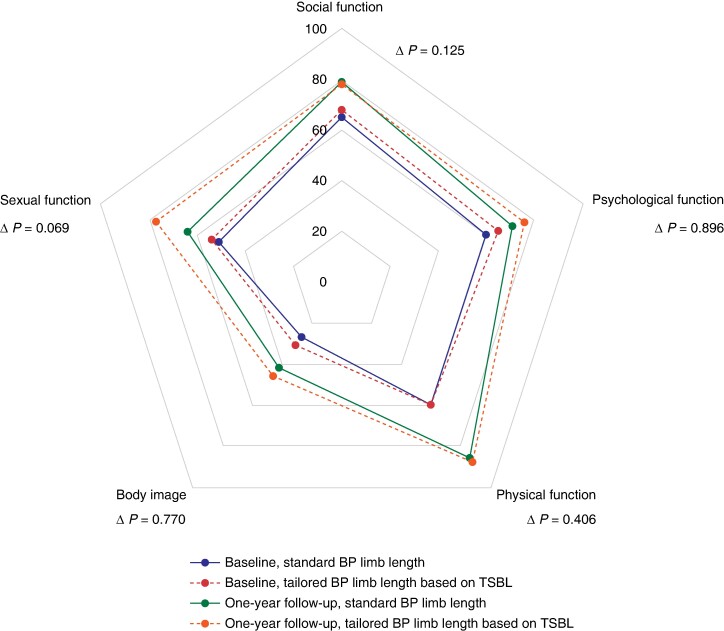
Outcomes of the questionnaire for patients with obesity (‘OBESI-Q’) regarding quality of life Values are mean scores. Group differences were assessed by comparing delta values, representing the differences between scores at 1-year follow-up and baseline. The questionnaire was assessed for 208 patients (missing data for 4 patients) at baseline and 198 patients (missing data for 14 patients) at 1-year follow-up. Sexual function was assessed for 145 patients (missing data for 67 patients) at baseline and 103 patients (missing data for 109 patients) at 1-year follow-up. BP, biliopancreatic; TSBL, total small bowel length.

Before surgery, the group with the tailored BP limb length had a higher proportion of patients with hypertension (37.4%) compared with the standard group (21.9%) (*P* = 0.014). After 1 year of follow-up, there were no differences observed in remission of hypertension, T2DM, and OSA between the two groups (*Fig. S1*). As shown in *Table 5*, at 1-year follow-up, daily bowel movements greater than or equal to five were reported by one patient (1.0%) in the standard BP limb group and no patients in the tailored BP limb group. At 1-year follow-up, early and late dumping, determined using the DSS, were present in 11.5% and 2.9% of the standard group and 12.4% and 5.7% of the tailored group respectively (*P* = 0.728 and *P* = 0.322 respectively). Reflux was reported by 7.7% of patients in the standard group and 5.7% of patients in the tailored group (*P* = 0.567). Quality of life increased significantly in both groups between baseline and 1-year follow-up (*P* < 0.001). The improvement in the five dimensions of quality of life did not differ between the groups at 1-year follow-up (*Fig. 3*).

**Table 5:** One-year complications for the intention-to-treat population

	Standard BP limb length, *n* = 104 (missing data *n* = 1)	Tailored BP limb length based on TSBL, *n* = 105 (missing data *n* = 2)	*P*
**Bowel movements at 1-year follow-up**			
Daily bowel movements ≥5	1 (1.0)	0 (0.0)	0.498
Number of days with >3 daily bowel movements in 2 weeks			
0	83 (79.8)	91 (86.7)	0.373
1–6	18 (17.3)	11 (10.5)	
7–10	3 (2.9)	3 (2.9)	
11–14	0 (0.0)	0 (0.0)	
Early dumping at 1-year follow-up	12 (11.5), *n* = 102 (missing data *n* = 3)	13 (12.4), *n* = 97 (missing data *n* = 10)	0.728
Late dumping at 1-year follow-up	3 (2.9), *n* = 102 (missing data *n* = 3)	6 (5.7), *n* = 97 (missing data *n* = 10)	0.322
Reflux	8 (7.7)	6 (5.7)	0.567
Use of PPI at 1-year follow-up	40 (38.5)	31 (29.5)	0.173
**Gastroscopy**	8 (7.7)	3 (2.9)	0.125
No abnormalities, *n*	4	2	
Gastritis, *n*	1	0	
Ulcer, *n*	2	1	
Stenosis, *n*	1	0	

Values are *n* (%) unless otherwise indicated. BP, biliopancreatic; TSBL, total small bowel length; PPI, proton pump inhibitor.

## Discussion

Tailoring the BP limb based on TSBL does not demonstrate superiority over a standard BP limb of 150 cm in terms of %TWL at 1 year. Tailoring BP limb length based on TSBL is a safe and feasible technique in terms of operating time and rates of short-term complications. The goal of the TAILOR study is to evaluate whether tailoring the BP limb length based on TSBL results in enhanced weight loss, while minimizing the risk of vitamin deficiencies. However, at 1-year follow-up, differences in weight loss outcomes are not observed; it is possible that disparities may become apparent in the longer term, which will require examination of the 3- and 5-year results of this study. Another explanation may be either that there is no difference or that variations in weight loss outcomes may only become apparent when employing even longer BP limb lengths, with more substantial differences between the lengths themselves (as lengths of 150, 180, and 210 cm are used in this study). By selecting these lengths, the percentage of bypassed bowel from the TSBL diminishes progressively. However, the authors opted not to exceed 250 cm based on evidence indicating a higher incidence of nutritional deficiencies with lengths of 250 cm^16^.

To the authors’ knowledge, there are only two other studies that have assessed tailoring the BP limb length based on TSBL in OAGB. Soong *et al*.^20^ tailored limb length based on preoperative BMI, increasing the BP limb by 10 cm for each BMI point, while measuring TSBL to keep the common channel at least 400 cm. They compared this technique in 470 patients with a retrospective matched cohort without TSBL measurements. At 1-year follow-up, they found no difference in weight loss, in agreement with the findings of the present study. Furthermore, they observed lower incidences of anaemia (5.9% *versus* 11.1%) and hypoalbuminaemia (1.5% *versus* 2.8%) in the TSBL measurement group. A possible explanation for the differences in nutritional outcomes is that they ensured a minimum common channel length of at least 400 cm. The incidence of deficiencies they observed in both groups is very low, despite using longer BP limb lengths (range 120–520 cm). It’s worth noting that their study compared the TSBL measurement group with retrospective data spanning 12 years, which could introduce variations in surgical techniques, postoperative care, follow-up, and patient selection, potentially influencing the observed differences. Komaei *et al*.^19^ investigated 64 OAGB patients with a fixed BP limb length of 200 cm and compared them with patients where 40% of the TSBL was bypassed (mean(s.d.) BP limb length of 250(44) cm). At 1-year follow-up, no significant difference in weight loss was reported, but the fixed BP limb group exhibited higher frequencies of deficiencies in vitamin A, vitamin D, and albumin. It is noteworthy that the fixed group had notably high rates of deficiencies, whereas the frequencies in the tailored group were comparable to the outcomes in both of the TAILOR study groups. Furthermore, it is important to consider the limitations of the study, including the small sample size, retrospective design, and the limited surgical experience of only 20 OAGB surgeries before the study, likely contributing to the variations in outcomes compared with the present study.

Several studies have shown that longer BP limb lengths are associated with increased weight loss outcomes in both OAGB and RYGB. At the same time, they are also linked to a higher incidence of nutritional deficiencies^14–18^. In a recent meta-analysis conducted by Salman *et al*.^15^, several OAGB studies comparing a BP limb length of 150 cm with a length of 200 cm were assessed. They revealed that a BP limb length of 200 cm led to superior weight loss, but also resulted in more severe nutritional deficiencies compared with a length of 150 cm. Similarly, an RCT conducted by Nergaard *et al*.^14^ focusing on RYGB showed that a longer BP limb length led to greater weight loss, but was also associated with more bowel movements and micronutrient deficiencies. Additionally, a systematic review by Zorrilla-Nunez *et al*.^18^ reported that, overall, RYGB patients with longer BP limbs experienced better weight loss outcomes. The existing literature on BP limb length in OAGB primarily comprises retrospective studies. The study by Ahuja *et al*.^16^ found more nutritional deficiencies at 1 year with a BP limb length of 250 cm compared with lengths of 150 and 180 cm when tailoring the BP limb length based on age, sex, BMI, co-morbidities, and diet. In the authors’ bariatric center, the authors showed, in a retrospective study, that adjusting the BP limb length based on baseline BMI did not abrogate the BMI differences after 3 years^36^.

Controversies exist surrounding the measurement of TSBL during gastric bypass surgery, as it has the potential to prolong the operating time and increase the risk of complications. However, in the present study, tailoring BP limb length based on TSBL did not result in significantly higher complication rates compared with patients who underwent OAGB without TSBL measurement in the authors’ routine practice. Only one case of intraoperative small bowel injury occurred, which was fixed by suturing during the operation, leading to no further complications. Additionally, the short-term complication rates are consistent with those reported in the existing literature^20,37–39^. TSBL measurement was feasible in 83% of the patients and required only a few minutes to perform. The mean duration of the operation, approximately 50 min, fell within the range of reported times found in the literature^8,39,40^.

In comparison with other studies, the incidence of hypoalbuminaemia is at the higher end of the reported mean values. Notably, there is considerable variability in reported prevalence rates (0–41%)^9,16,41,42^. This variability may be attributed in part to differences in the thresholds used to define hypoalbuminaemia (albumin less than 25–35 g/l), with no clear consensus on the most clinically relevant cut-off^40,42^. Furthermore, proton pump inhibitors (PPIs) were prescribed at a low threshold, guided by the authors’ observation that PPIs have a positive impact on alleviating complaints, thereby explaining the disparity in percentages between PPI use and reported reflux.

This RCT has limitations. First, the study was not powered to detect differences in the secondary outcomes of nutritional deficiencies and no firm conclusions can be drawn based on nutritional deficiencies similarly to all other secondary outcomes. Second, the exclusion of BMI greater than 50 kg/m^2^ and preoperative vitamin deficiencies limits generalizability, in addition to the single-centre design. The definition of T2DM remission lacks clarity regarding the duration of normal or lowering HbA1c values and the duration of cessation or reduction of medication^32^. Another limitation is the absence of preoperative weight loss data following recent IFSO consensus guidelines as the preoperative weight used in this study was the weight on the day of the operation^43^. The hand-over-hand method used for TSBL measurement relies on surgeon estimation of 5 cm increments and therefore is not a precise measurement method. However, a study by the authors was conducted to assess the variability of bariatric surgeons, revealing a mean deviation of less than 10%^27^. Thus, despite any variability in the intended BP limb lengths of 150, 180, and 210 cm in the present study, it is unlikely that the groups significantly overlap. The shortest recorded TSBL of 295 cm in the present study appears relatively short compared with bowel lengths reported in some bariatric research papers^19,20,25^. Nonetheless, comparable lengths have been reported in other studies, emphasizing the potential influence of different TSBL measurement methods^44,45^. The present study has strengths in the randomized double-blind design, high follow-up rate, and comprehensive data collection. To the authors’ knowledge, this is the first RCT on tailoring BP limb length based on TSBL in OAGB. A major limitation is that this predefined 1-year analysis is a very short follow-up, preventing any conclusions at this point and the primary endpoint results at 5 years are needed to address the study hypothesis. Nevertheless, reporting the predefined assessment point at 1 year provides valuable insights into the feasibility and safety of the technique.

In conclusion, tailoring BP limb length based on TSBL is a safe and feasible technique. Tailoring the BP limb based on TSBL is not superior to a standard BP limb in terms of %TWL at 1 year.

## Supplementary Material

znae219_Supplementary_Data

## Data Availability

The data sets analysed and the statistical codes are available from the corresponding author on reasonable request.
