# Supplementary material for: Effect of tailoring biliopancreatic limb length based on total small bowel length versus standard limb length in one anastomosis gastric bypass: 1-year outcomes of the TAILOR randomized clinical superiority trial
Source: Br J Surg. 2024 Aug 30;111(9):znae219. doi: 10.1093/bjs/znae219 (PMC11363871; doi:10.1093/bjs/znae219)
Supplement: znae219_Supplementary_Data [file znae219_supplementary_data.zip › Supplementary file 1 - studie protocol TAILOR .docx]

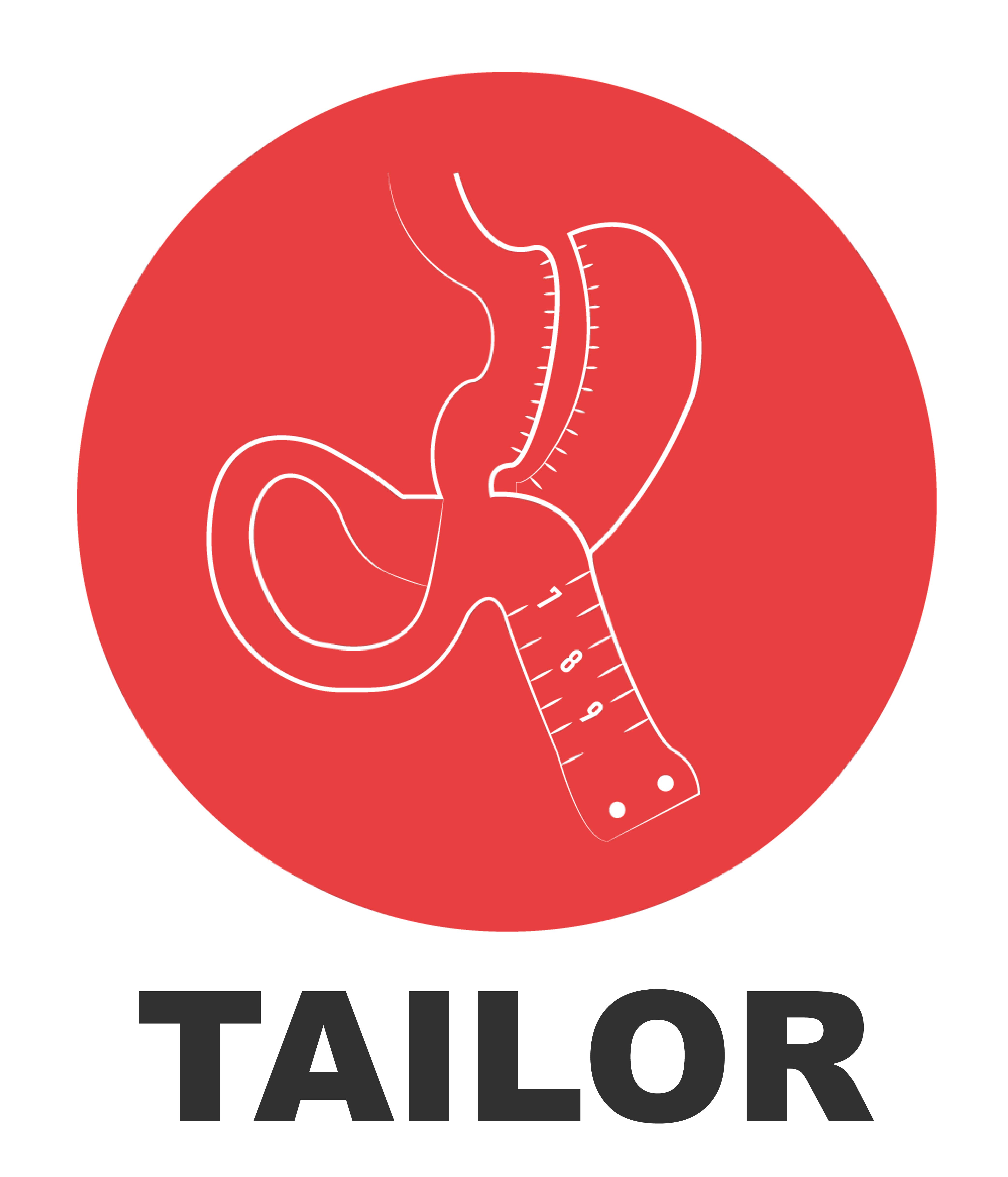


**PROTOCOL TITLE** :

**TA**iloring L**I**mb length based on total small bowel **L**ength

in **O**mega-loop gastric bypass su**R**gery : the **TAILOR** study

amendment 1

21-jan-2020

Tailoring Limb length based on total small bowel length in omega-loop gastric bypass

| **Protocol ID** | **CON-VOLUME 004** |
| --- | --- |
| **Short title** | Limb Length OLGB based on small bowel length |
| **EudraCT number** | nvt |
| **Version** | 1 |
| **Date** | 12-09-2019 |
| **Coordinating investigator/project leader** | ***nvt*** |
| **Principal investigator(s) (in Dutch: hoofdonderzoeker/ uitvoerder)**  ***<Multicenter research: per site>*** | L.J.M. de Heide, MD, Internist-Endocrinoloog, Centrum Obesitas Noord-Nederland ( CON)/ Medisch Centrum Leeuwarden  nvt |
|  |  |
| **Sponsor (in Dutch: verrichter/opdrachtgever)** | Medisch Centrum Leeuwarden |
|  | **KVK: 01137890** |
| **Subsidising party** | FitForMe BV, Rotterdam, KVK: 24375270  Stichting CON-VOLUME research, KVK : 72036001 |
| **Independent expert (s)** | Dr.R.Gerritsen  Internist-Intensivist  Medisch Centrum Leeuwarden  31-582866666 |
| **Laboratory sites <*if applicable*>** | Certe , Medische diagnostiek & advies, Groningen |
|  |  |
| **Pharmacy <*if applicable*>** | nvt |
|  |  |

**PROTOCOL SIGNATURE SHEET**

| **Name** | **Signature** | **Date** |
| --- | --- | --- |
| **Sponsor or legal representative:**  **Medisch Centrum Leeuwarden**  **Head of Department:**  **M.Emous, surgeon, CON** |  |  |
| **[Coordinating Investigator/Project leader/Principal Investigator]:**  **L.J.M. de Heide, internist-endocrinologist, CON** |  |  |

**TABLE OF CONTENTS**

1. INTRODUCTION AND RATIONALE 8

2. OBJECTIVES 9

3. STUDY DESIGN 10

4. STUDY POPULATION 11

4.1 Population (base) 11

4.2 Inclusion criteria 11

4.3 Exclusion criteria 11

4.4 Sample size calculation 11

5. TREATMENT OF SUBJECTS 12

5.1 Investigational product/treatment 12

6. NON-INVESTIGATIONAL PRODUCT 14

6.1 Name and description of non-investigational product(s) 14

6.2 Summary of findings from non-clinical studies 14

6.3 Summary of findings from clinical studies 14

6.4 Summary of known and potential risks and benefits 14

6.5 Description and justification of route of administration and dosage 14

6.6 Dosages, dosage modifications and method of administration 14

6.7 Preparation and labelling of Non Investigational Medicinal Product 14

6.8 Drug accountability 14

7. METHODS 15

7.1 Study parameters/endpoints 15

7.1.1 Main study parameter/endpoint 15

7.1.2 Secondary study parameters/endpoints (if applicable) 15

7.1.3 Other study parameters (if applicable) 15

7.2 Randomisation, blinding and treatment allocation 15

7.3 Study procedures 15

7.4 Withdrawal of individual subjects 15

8.4.1 Specific criteria for withdrawal (if applicable) 15

7.5 Replacement of individual subjects after withdrawal 15

7.6 Follow-up of subjects withdrawn from treatment 15

7.7 Premature termination of the study 15

8. SAFETY REPORTING 16

16

8.2 AEs, SAEs and SUSARs 16

8.2.1 Adverse events (AEs) 16

8.2.2 Serious adverse events (SAEs) 16

8.2.3 Suspected unexpected serious adverse reactions (SUSARs) 17

8.3 Annual safety report 18

8.4 Follow-up of adverse events 19

8.5 [Data Safety Monitoring Board (DSMB) / Safety Committee] 19

9. STATISTICAL ANALYSIS 20

9.1 Primary study parameter(s) 20

9.2 Secondary study parameter(s) 20

9.3 Other study parameters 20

9.4 Interim analysis (if applicable) 20

10. ETHICAL CONSIDERATIONS 21

10.1 Regulation statement 21

10.2 Recruitment and consent 21

10.3 Objection by minors or incapacitated subjects (if applicable) 21

10.4 Benefits and risks assessment, group relatedness 21

10.5 Compensation for injury 21

10.6 Incentives (if applicable) 22

11. ADMINISTRATIVE ASPECTS, MONITORING AND PUBLICATION 23

11.1 Handling and storage of data and documents 23

11.2 Monitoring and Quality Assurance 23

11.3 Amendments 23

11.4 Annual progress report 24

11.5 End of study report 24

11.6 Public disclosure and publication policy 25

12. STRUCTURED RISK ANALYSIS 26

12.1 Potential issues of concern 26

12.2 Synthesis 27

13. REFERENCES 27

**LIST OF ABBREVIATIONS AND RELEVANT DEFINITIONS**

| **ABR** | **General Assessment and Registration form (ABR form), the application form that is required for submission to the accredited Ethics Committee; in Dutch: Algemeen Beoordelings- en Registratieformulier (ABR-formulier)** |
| --- | --- |
| **AE** | **Adverse Event** |
| **AR** | **Adverse Reaction** |
| **CA** | **Competent Authority** |
| **CCMO** | **Central Committee on Research Involving Human Subjects; in Dutch: Centrale Commissie Mensgebonden Onderzoek** |
| **CV** | **Curriculum Vitae** |
| **DSMB** | **Data Safety Monitoring Board** |
| **GCP** | **Good Clinical Practice** |
| **GDPR** | **General Data Protection Regulation; in Dutch: Algemene Verordening Gegevensbescherming (AVG)** |
| **IB** | **Investigator’s Brochure** |
| **IC** | **Informed Consent** |
| **METC** | **Medical research ethics committee (MREC); in Dutch: medisch-ethische toetsingscommissie (METC)** |
| **(S)AE** | **(Serious) Adverse Event** |
|  |  |
| **Sponsor** | **The sponsor is the party that commissions the organisation or performance of the research, for example a pharmaceutical**  **company, academic hospital, scientific organisation or investigator. A party that provides funding for a study but does not commission it is not regarded as the sponsor, but referred to as a subsidising party.** |
| **SUSAR** | **Suspected Unexpected Serious Adverse Reaction** |
| **UAVG** | **Dutch Act on Implementation of the General Data Protection Regulation; in Dutch: Uitvoeringswet AVG** |
| **WMO** | **Medical Research Involving Human Subjects Act; in Dutch: Wet Medisch-wetenschappelijk Onderzoek met Mensen** |

**SUMMARY**

**Rationale:**

Omega-loop gastric bypass (OLGB) surgery aims to reduce weight in obese patients to a healthy BMI. Surgeons usually creat a standard length of the biliopancreatic limb (BP-limb) based on own experience. This practice leads in a substantial percentage of patients to either persisting obesity or underweight. Furthermore, a substantially percentage of patients develop disturbing diarrhea and vitamin and mineral deficiencies. The total small bowel length (TSBL) varies in subjects between less than 5 to more than 10 meters. This variance could have consequences both for weight loss and deficiencies after OLGB. The aim of the study is to investigate whether adjusting the length of the BP-limb tot the TSBL leads to more weight loss with smaller variance and to less bowel movements and less deficiencies of vitamins and minerals using an optimal multivitamin Fitforme WSL primo.

**Objective**:

Primary :

To compare the percent total weight loss (%TWL) at 5 years between the group with the standard BP-length and the group with an adjusted BP-length.

Secondary :

To compare the proportion of patients with a BMI between 22 and 30 Kg/m^2^,the mean number of daily bowel movements and number of days with daily bowel movements > 3 over in the last two weeks before a visit, Quality of life measured by the RAND-36 questionnaire between the groups and the percentage of patients experiencing moderate to severe dumping symptoms defined by the Dumping Severity Score (DSS) between the groups.

To compare the proportion of patients in the two groups who have neither deficiencies of iron, nor vit. D, nor vit B12 without extra suppletion, during the study

**Study design:** double blind intervention study

**Study population:**

Patients between 18 and 65 years with obesity class III or II with comorbidity (based on the IFSO criteria and Dutch guideline on obesity), scheduled for OLGB surgery and willing to participate.

**Intervention** :

OLGB : patients will be randomly allocated to either a standard BP-limb of 150 cm or to a BP-limb length based on their TSBL : TSBL < 500 cm : BP-limb : 150 cm ; TSBL 500-700 cm : BP-limb 180 cm ; TSBL > 700 cm : BP-limb : 210 cm.

**Main study parameters/endpoints:**

Percent total weight loss (%TWL) at 5 years.

Proportion of patients with 22 ≤ BMI ≤ 30 Kg/m^2^

Mean number of daily bowel movements in the last two weeks

Mean number of days with daily bowel movements > 3 in the last two weeks

Quality of life measured by the RAND-36 questionnaire

Percentage of patients experiencing moderate to severe dumping symptoms defined by the Dumping Severity Score (DSS)

Percentage of patients who have neither deficiencies of iron, nor vit. D, nor vit B12 without extra suppletion

**Nature and extent of the burden and risks associated with participation, benefit and group relatedness:**

Measuring TSBL will take 10 minutes of extra time during the surgery and increases slightly the risk of damaging the intestine. Bariatric surgeons are experienced in measuring small bowel. Patients have 4 extra visits in 5 years and during routine blood testing an extra bloodsample of 20 ml will be drawn for research purposes. They have to fill in 2 questionnaires at their visits. Benefit is a free providing of a daily multivitamin capsule during the study and possibly a better outcome after surgery in terms of weight loss and side effects.

# INTRODUCTION AND RATIONALE

Omega-loop gastric bypass, also known as mini-gastric bypass (OLGB/MGB) is currently one of the most effective treatment options for morbid obesity. It is technically a more simple procedure compared to the RYGB and has proven in some studies to have a better outcome in terms of weight loss and reduction of co-morbidities.

The aim of the weight loss surgery is to achieve an optimum weight loss aiming at a BMI of 25 kg/m^2^ in combination with a minimum of side effects, like vitamin, macronutrient, mineral deficiencies and diarrhea.

There is substantial variation in the determination of the biliopancreatic (BP) limb length with some surgeons using a fixed length and some a length based on the initial BMI. The length varies between less than 150 cm to more than 250 cm. A recent retrospective study by Charalampos et.al. adjusting limb length from 200 to 300 cm depending on BMI resulted in on average comparable EWL when corrected for initial BMI. The average BMI after 36 months was 27.5 Kg/m2 (± 5.3). This strategy of BP based on initial BMI results in 16% of patients with a final BMI > 33 and 16% with less than 22.

Furthermore despite the advice to take life-long (over-the-counter) multivitamin suppletion 20% or more patients developed de novo deficiencies of iron, vit B12, vit D, and/or minerals in the first postoperative year (1).

Ahuja et.al. adjusted the limb length from 150 to 250 cm depending on BMI and also found increasing number of deficiencies of micro-nutrients with increasing BP length (2). In this study patients were advised to use a multivitamin supplement, however no specific brand was used.

Some evidence exists in RYGB surgery that the length of the BP-limb influences weight loss. Nergaard et al. performed a RCT comparing a RYGB with a BP-limb of 200 to 60 cm with a RYGB with an alimentary limb of 60 and 150 cm respectively. The longer BP-limb length led to more weight loss but also to more bowel movements and micronutrient deficiencies (3). Total small bowel length was measured in their whole patient population and varied between 480 and 870 cm but was not taken in to account in terms of stratification.

Zorilla et.al performed a systematic review on limb length in RYGB and found 13 studies meeting adequate quality (4). Weight loss on the whole was better in patients with longer BP limbs.

Earlier studies showed that the length of the common channel plays a role in the outcome of surgery both in weight loss and in micronutrient deficiencies. The length of the total small bowel can vary considerably with measured values between 350 to more than 1000 cm (5).

In this study of Tacchino in 443 patients TSB median length was 690 cm (women 678±92, men 728±85 cm) with a SD of 94 cm. In males 3% had a bowel length < 400 cm and 15% > 800, in females 2% had a length of < 400 cm and 5% > 800 cm.

In current practice in OLGB surgery surgeons use a fixed BP-limb length or adjust the length to the initial BMI. Total weight loss varies in literature between 30-35 % with SD up to 8.5%, aiming at a BMI result between 23 and 30 Kg/m^2^

The contribution of the length of the residual small bowel, total minus the BP-limb, on weight loss and deficiencies has up till now not been studied in OLGB surgery.

# OBJECTIVES

To investigate whether adjusting the length of the BP-limb of the OLGB based on measured total small bowel length leads to more weight loss, resolution of co-morbidities with less development of micronutrient deficiencies and bowel movements compared a to a standard limb length in OLGB in patients using a standardized multivitamin supplement (FitForMe Primo).

**Primary Objective**:

To compare the percent total weight loss (%TWL) at 5 years between the group with the standard BP-length and the group with a adjusted BP-length .

**Secondary Objective(s):**

1) To compare the percent of total weight loss (%TWL) between the groups at 1,2,3 and 4 years.

2) To compare the percent excess weight loss (%EWL) between the groups at 1,2,3, 4 and 5 years.

Definition: %EWL (Initial Weight – Postop Weight) / (Initial Weight – Ideal Weight)

(ideal weight is defined by the weight corresponding to a BMI of 25 kg/m2 )

3) To compare the percent excess BMI loss (%EBMIL) between the groups at 1,2, 3, 4 and 5 years.

Definition: %EBMIL (ΔBMI / (Initial BMI – 25)) * 100)

4) To compare the proportion of patients with 22 ≤ BMI ≤ 30 between the groups at 1,2 3, 4 and 5 years.

5) To compare the mean number of daily bowel movements and number of days with daily bowel movements > 3 in the last two weeks, between the groups at 6 months, 1,2, 3, 4, and 5 years.

6) To compare the Quality of life measured by the RAND-36 questionnaire between the groups at 6 months, 1,2, 3, 4 and 5 years, absolute and compared to before surgery

7) To compare the percentage of patients experiencing moderate to severe dumping symptoms defined by the Dumping Severity Score (DSS) (see Emous et al.) at 6 months, year 1, 2, 3, 4 and 5 between the groups, absolute and compared to before surgery.

8) To compare the proportion of patients in the two groups who have neither deficiencies of iron, nor vit. D, nor vit B12 without extra suppletion, at 6 months, 1, 2, 3, 4 and 5 years. Deficiencies are defined by the lower border of the local normal laboratory values.

9) To compare the proportion of patients in the two groups who have a deficiency at 6 months, 1, 2, 3, 4 and 5 years, or received extra suppletion for :

- Vitamin B1

- Vitamin B6

- Folic acid

- Vitamin A

- Vitamin D

- Calcium

- Phosphate

- Albumin

- Zinc

- Copper (visit 1,3,5 years)

- Selenium (visit 1,3,5 years)

Deficiencies are defined by the lower border of the local normal laboratory values.

10)To compare the proportion of patients between the groups at 1, 2, 3, 4 and 5 years without diabetes, both those with remission of diabetes and those without de novo diabetes, defined as an HbA1c less than 48 mmol/mol without diabetes medication in the last 6 months. ^#^

11) To compare the proportion of patients between the groups at 1, 2, 3,4 and 5 years with improvement of diabetes, defined as a reduction of HbA1c with at least 10 mmol/mol, but not reaching remission criteria and/or less anti-diabetic medication ^#^.

12) To compare the proportion of patients between the groups at 1, 2, 3, 4 and 5 years with remission of hypertension, defined as a blood pressure of 140/90 mmHg or less without antihypertensive medication ^#^.

13) To compare the proportion of patients between the groups at 1, 2, 3, 4 and 5 years with improvement of hypertension, defined as a lower blood pressure of at least 10 mm Hg systolic and/or 5 mmHg diastolic, and/or less antihypertensive medication (not reaching the criterion of remission) ^#^.

14) To compare the proportion of patients between the groups at 1, 2, 3, 4 and 5 years with resolution of sleep apnea, defined by cessation of CPAP or other devices use, documented by their own pulmonologist

15) To perform analysis of the above parameters in the subgroups with TSBL 500-700 and >700.

16) To compare within the standard group of patients with an allocated BP-limb length of 150 cm those with a TSBL < 500 to those with a TSBL 500-700 and to those with TSBL >700 cm on the above mentioned parameters.

^#^ : patients are asked to bring along with their visit an overview of current medication from their local pharmacist and actual use will be checked with the patient. In case of absence of the overview, after informed consent is given, the local pharmacist will be asked to provide the investigator with an overview of the medication

# STUDY DESIGN

This is a double-blind randomized 5 year study with two arms. Patients who are scheduled for primary OLGB surgery are eligible when during the surgery total small bowel length can be measured and when they are able to swallow the Fitforme WLS primo multivitamin capsule. Patients will be allocated to one of the two surgical treatment arms by randomization at the beginning of the operation. Only the operating surgeon is aware of the allocation and will document total small bowel length, treatment allocation and BP-length in a coded database. Both the TSBL and the length of the BP-limb will not be documented in the patient file. The investigators and the patient will not be informed on the BP-limb length during the study.

Patients with a small bowel that could not be measured during surgery will be treated according to the current daily practice and will not enter the study.

# STUDY POPULATION

## Population : patients from the Centrum Obesity North-Netherlands (CON), who comply with the IFSO criteria during a multidisciplinary screening and have chosen to have a primary mini-GB (OLGB) and are willing to participate.

## Inclusion criteria

In order to be eligible to participate in this study, a subject must meet the following criteria:

- Age between 18 and 65
- BMI > 40 kg/m^2^ or
- BMI > 35 kg/m^2^  with co-morbidity : diabetes, hypertension, OSAS, arthrosis
- Willing to participate with written informed consent before start of the surgery
- A completely measured total small bowel during surgery
- No pre-operative deficiencies of vit B_12_, vit D and iron, measured with ferritin
- No use of extra (multi-)vitamin supplements with exception of vit D max 800 IU/day
- Able to swallow the multivitamin FFM WLS primo (tested before surgery)

## Exclusion criteria

A potential subject who meets any of the following criteria will be excluded from participation in this study:

- BMI > 50 Kg/m^2^
- Known gastro-intestinal disease or history of gastro-intestinal disease, e.g. celiac disease, inflammatory bowel disease
- Known addiction behaviour
- Intolerance to Fitforme Primo multivitamin
- Pregnancy planning within the first two years after surgery
- Renal or hepatic insufficiency
- Former abdominal surgery unabling the measurement of the small bowel during laparoscopic surgery
- Parenteral use of vit B_12_

## Sample size calculation

The null hypothesis in the study is as follows : the percentage TWL at five years in both groups is not different with a mean of 35. The alternative hypothesis is that the mean of the absolute difference in TWL in the experimental group is 5% more than the standard group (40% and 35% respectively).

For the evaluation of the primary endpoint of the percent total weight loss (%TWL) at five years, a sample size of 2x 92 will achieve 80% power to detect a (absolute) difference of -5.0% between the null hypothesis ( both group means of %TWL are 35.0) and the alternative hypothesis (mean of experimental group is a %TWL of 40.0) with estimated group standard deviations of 12.0 and 12.0 and with a significance level (alpha) of 0.05 using a two-sided two-sample t-test.

In case of a reduced standard deviation in the active treatment group of 9.0, the power achieved is 88.8%.

With an expected maximum drop-out due to conversion of OAGB to RYGB of 15% the sample size is 92 plus 14 = 106 patients in each group, leading to a total of 212 participants in the study.

# TREATMENT OF SUBJECTS

Patients will be randomly allocated to one of the two treatment arms :

- 1. A standard BP-limb length of 150 cm
- 2. A BP-limb length depending on total small bowel length (TSBL) measured during the surgical procedure :
- TSBL : < 500 cm : 150 cm
- TSBL : 500-700 cm : 180 cm
- TSBL : > 700 cm : 210 cm

## Summary of findings from non-clinical studies

In diabetic rats long limb duodenal-jejuno-bypass (DJB) results in more weight loss than standard DJB (Zhang). No other studies are available.

## Summary of findings from clinical studies

In RYGB surgery studies have suggested that the length of the common channel can influence the outcome of weight loss, however no studies have been performed using the total small bowel length as a criterion for length of bypass, neither in RYGB nor in OLGB.

Adjusting of BP-limb length based on the initial BMI in OLGB has been done resulting in more weight loss with more BP-length however with also more vitamin and micronutrient deficiencies (2)

##

## Summary of known and potential risks and benefits

Measuring total small bowel length induces a slightly increase in risk of damaging the bowel compared to just measuring the length of the BP-limb. Surgeons are experienced in measuring bowel length in daily practice. This measurement will increase time of surgery with on average 10 minutes.

In OLGB surgery surgeons use BP-limb lengths varying between 150 - 250 cm based on personal experience. The different lengths in our study are within these measures. Surgical risks are not higher when BP-limb length is increased. Potential risks are of course to much or to little weight loss and more than expected deficiencies of vitamins and micronutrients.

Potential benefits are a better outcome of weight reduction compared to current usual care without increase in vitamin and/or micronutrient deficiencies.

# NON-INVESTIGATIONAL PRODUCT

## Name and description of non-investigational product(s)

Fitforme WSL Primo is a multivitamin and mineral containing product with a content that is especially developed for patients after OLGB. To increase compliance, it is given as a once daily dose.

## Summary of findings from non-clinical studies

Not available

## Summary of findings from clinical studies

A RCT comparing WLS Primo to conventional multivitamin supplements is currently underway (Prager et.al).

## Summary of known and potential risks and benefits

There are no known risks involved in multivitamin suppletion with the preparation used. Especially, vitamin A, B1 and vitamin D are within the range defined for safe use. Benefits are the prevention of development of vitamin and mineral deficiencies

## Description and justification of route of administration and dosage

The advised dose is one capsule once daily, orally administrated.

## Dosages, dosage modifications and method of administration

N.a.

## Preparation and labelling of Non Investigational Medicinal Product

FitForMe WSL Primo will be delivered by the regular manufacturer and distributed within their own packaging by the research team.

## Drug accountability

Each patient will be given a standard amount of the multivitamin, for the time to the next visit plus 30 extra days. They are advised to bring back all the multivitamin capsules left as well as the residual empty blisters and boxes at the day of the visit in which their number of pills are filled out to the amount for the next period plus for 30 days extra. By counting the left over pills and substracting them from the number handed out in the former visit accountability can be checked.

# METHODS

## Study parameters/endpoints

### Main study parameter/endpoint

Percent total weight loss (%TWL) at 5 years.

Assumptions : Control group : TWL of 35% (SD 12%)

Experimental group : TWL of 40% (SD 12 or 9%)

(Absolute difference TWL 5% with possible smaller SD)

Definition : TWL = ([initial weight – attained weight]/ initial weight) * 100

### Secondary study parameters/endpoints

1) Percent of total weight loss (%TWL) at 1,2,3,and 4 years.

2) Percent excess weight loss (%EWL) at 1,2,3, 4 and 5 years.

Definition: %EWL (Initial Weight – Postop Weight) / (Initial Weight – Ideal Weight)

(ideal weight is defined by the weight corresponding to a BMI of 25 kg/m2 )

3) Percent excess BMI loss (%EBMIL) at 1,2,3, 4 and 5 years.

Definition: %EBMIL (ΔBMI / (Initial BMI – 25)) * 100]

4) Proportion of patients with 22 ≤ BMI ≤ 30 Kg/m^2^ at 1,2,3, 4 and 5 years

5) Mean number of daily bowel movements in the last two weeks at 6 months, 1,2,3, 4 and 5 years.

6) Mean number of days with daily bowel movements > 3 in the last two weeks at 6 months, 1,2,3, 4 and 5 years.

6) Quality of life measured by the RAND-36 questionnaire at 6 months, 1,2,3, 4 and 5 years, absolute and compared to before surgery.

7) percentage of patients experiencing moderate to severe dumping symptoms defined by the Dumping Severity Score (DSS) at 6 months, 1,2,3, 4 and 5 years, absolute and compared to before surgery.

Moderate to severe is defined as 3 or more dumping symptoms with a severity score of 2 or 3 on a 4-point Likert scale. Symptoms are divided in two categories, e.g. early and late,

as described by Emous et.al. (7)

8) percentage of patients who have neither deficiencies of iron, nor vit. D, nor vit B12 without extra suppletion, at 6 months, 1,2,3, 4 and 5 years . Deficiencies are based on the local normal laboratory values.

9) Proportion of patients who have a deficiency at 6 months, 1,2,3, 4 and 5 years, or received extra suppletion for:

- iron (ferritin)

- vitamin B_12_

- vitamin D_3_

- Vitamin B_1_

- Vitamin B_6_

- Folic acid

- Vitamin A

- Vitamin D

- Calcium

- Phosphate

- Albumin

- Zinc

- Copper at 1,3 or 5 years

- Selenium at 1,3 or 5 years

10)Proportion of patients at 1,2,3, 4 and 5 years without diabetes, both with remission and without de novo, defined as an HbA1c less than 48 mmol/mol without diabetes medication in the last 6 months.

11)Proportion of patients at one year and two years with improvement of diabetes, defined as a reduction of HbA1c of 10mmol/mol or more but not reaching remission criteria and/or less anti-diabetic medication.

12)Proportion of patients at 1,2,3, 4 and 5 years with remission of hypertension, defined as a blood pressure of 140/90 mmHg or less without antihypertensive medication.

13)Proportion of patients at 1,2,3, 4 and 5 years with improvement of hypertension, defined as a lower blood pressure and/or less antihypertensive medication (not reaching the criterion of remission).

14)Proportion of patients at 1,2,3, 4 and 5 years with resolution of sleep apnea, defined by cessation of CPAP or other devices use, documented by their own pulmonologist

### Other study parameters

Measurements of CRP, hemoglobin, WBC, thrombocytes, sodium, potassium, creatinine, BUN, ASAT, ALAT, alkaline phosphatase, yGT, HbA1c are part of routine investigation in the follow-up and will also be part of the study

Serum will be stored for analysis in later stage in case of new developments or insights relevant for the study become evident.

## Randomisation, blinding and treatment allocation

After informed consent is given, the patient is asked to swallow one Fitforme WSL primo capsule. A patient who is not able to swallow the capsule will be withdrawn form the study.

Randomization is performed using a table of random figures delivered by a independent party (clinical pharmacist), using envelopes with the treatment allocation.

The envelope contains a study number on the outside and the same number with one of the treatment arms within. The envelope will be opened by the OR nurse when the surgeon has succeeded in measuring the TSBL.

If the surgeon is not able to measure the TSBL the patient will be withdrawn from the study.

The study number and the patients medical file number will be forwarded to the principle investigator by the surgeon.

The surgeon will document the allocation, total small bowel length and length of the BP-limb in the file containing only study numbers. In the follow-up neither any of the usual care medical providers nor the patient is aware of the allocation or BP-limb length. The surgeon will not describe the TSBL and the BP-limb length in his surgical report.

## Study procedures

A standard operation protocol for OLGB will be used in all patients. The pouch will be made with the help of a linear stapler. At first, the stomach will be transected horizontally at the junction of the corpus and antrum at the level of crow’s foot. A small pouch is made with help of a calibration sonde of 34 French, the pouch is finished near the angle of His. Then the total limb length is measured. With a succesfull TSBL measurement the OR nurse will open the envelop and the allocated procedure will be communicated to the surgeon. Based on the randomization outcome biliopancreatic limb length is measured. In case the measurement of the total limb length does not succeed, standard limb length of 150 cm is performed. An antecolic, retrogastric anastomosis is performed by liniar stapling and the remaining defect is closed by V-loc. An anti-reflux stitch is performed. Integrity of the anastomoses is checked by methylene-blue-test and in case of leakage, extra sutures will be applied. The mesenterial defect of Peterson will be closed as far as possible by endo hernia stapling or unresolvable V-loc.

The extra measurements of limb length will take less than ten minutes of time. In patients allocated to standard treatment the surgeon will construct a BP-limb of 150 cm. In patients allocated to the adjustment length, the length of the BP-limb will depend on the total small bowel length : < 500 cm : 150 BP; 500-700 cm : 180 BP and > 700 cm : 210 BP.

On leave of the hospital the patients will be provided with Fitforme WSL Primo multivitamin capsules for the following period and will be instructed to use one capsule once daily.

Follow-up will take place in accordance with usual care, i.e. after 1,6,12 and 24 months and yearly up till 5 years. Extra visits will be at 18,30,42 and 54 months for compliance check and distribution of FFM multivitamins.

Weight measurements and blood samples will be taken in accordance with the standard protocol of follow-up after bariatric surgery in our institute added with 20 ml for selenium and copper measurements and future lab tests at 1,3 and 5 years.

Data from 6,12 and 24 months and every year thereafter up to five years after surgery will be anonymously collected in a database.

At each visit standard care includes :

- inquiry after well being and complaints, side effects, adverse events
- documenting current medication use, both regular and over-the-counter, and when applicable CPAP use
- inquiring after co-morbidities
- measurement of body weight, blood pressure and pulse
- drawing of blood samples for routine post operative care including :

CRP, hemoglobin, WBC, thrombocytes, sodium, potassium, creatinin, BUN, ASAT, ALAT, alkaline phosphatase, yGT, HbA1c, glucose, calcium, magnesium, phosphate, albumin, vit B_1_, vit B_6_, vit B_12_, folic acid, ferritin, vit A, vit D, Zinc

- Scheduling a new appointment

If the patient forgot to bring along a recent overview of current medication from their own pharmacy , the actual medication use will be requested from the local pharmacy.

Extra effort for the patient in the study includes :

- Bringing along a written overview of current medication from the local pharmacy to the appointment.
- Bringing back all used and non-used blisters and boxes for counting of compliance of the use of the FitForMe WLS primo pills
- filling in the DSS questionnaire
- filling in the RAND 36 questionnaire
- drawing of 20 ml of extra blood during the venepuncture for selenium, copper and storage for later investigations at 1,3 and 5 years
- Visits at 18, 30,42, and 54 months for counting left over pills and distributing new ones for the forthcoming 6 months

All patients will be treated with 800 IU vitamin D and 500 mg Calcium in a combination pill as part of standard care.

In case of deficiencies of vitamins or micronutrients they will be substituted according to standard care.

## Withdrawal of individual subjects

Subjects can leave the study at any time for any reason if they wish to do so without any consequences. The investigator can decide to withdraw a subject from the study for urgent medical reasons.

### Specific criteria for withdrawal

Patients who are not able to swallow the FFM WSL primo capsule and those in whom the surgeon cannot measure the TSBL will be withdrawn from the study and will be replaced.

Patients who develop complications during the first 30 days needing revisional surgery changing bowel length will be withdrawn from the study.

Patients who need a conversion from the OLGB to a RYGB during the study will be withdrawn from the study and not replaced. They will be analysed on a intention to treat principle and all data up to the conversion will be used in the study.

## Replacement of individual subjects after withdrawal

Patients withdrawn because of problems with swallowing the FFM WLS primo pill, no measurement of TSBL, or those withdrawn for other reasons in the first 30 days will be replaced.

## Follow-up of subjects withdrawn from treatment

Patients who are withdrawn from the study remain in usual care

## Premature termination of the study

Reasons for prematurely termination of the study are :

an unexpectedly high rate of surgical complications due to measuring total small bowel length or adjusting the BP-limb length as to the discretion of the investigator.

An unacceptable percentage of complications like excessive weight loss, diarrhea or nutrient deficiencies in either study arms as to the discretion of the investigator .

This will be investigated, at first without unsealing treatment allocation after the first 50 and 100 operated patients (operative complications) and after 1 and 2 years after start of the study for non-operative complications. If there is a high complications rate in the whole group treatment allocation will be unsealed to investigate further.

# SAFETY REPORTING

## Temporary halt for reasons of subject safety

In accordance to section 10, subsection 4, of the WMO, the sponsor will suspend the study if there is sufficient ground that continuation of the study will jeopardise subject health or safety. The sponsor will notify the accredited METC without undue delay of a temporary halt including the reason for such an action. The study will be suspended pending a further positive decision by the accredited METC. The investigator will take care that all subjects are kept informed.

## AEs, SAEs and SUSARs

### Adverse events (AEs)

Adverse events are defined as any undesirable experience occurring to a subject during the study, whether or not considered related to the experimental intervention. All adverse events reported spontaneously by the subject or observed by the investiga­tor or his staff will be recorded.

### Serious adverse events (SAEs)

A serious adverse event is any untoward medical occurrence or effect that

- results in death;
- is life threatening (at the time of the event);
- requires hospitalisation or prolongation of existing inpatients’ hospitalisation;
- results in persistent or significant disability or incapacity;
- is a congenital anomaly or birth defect; or
- any other important medical event that did not result in any of the outcomes listed above due to medical or surgical intervention but could have been based upon appropriate judgement by the investigator.

An elective hospital admission will not be considered as a serious adverse event.

The investigator will report all SAEs to the sponsor without undue delay after obtaining knowledge of the events.

The sponsor will report the SAEs through the web portal *ToetsingOnline* to the accredited METC that approved the protocol, within 7 days of first knowledge for SAEs that result in death or are life threatening followed by a period of maximum of 8 days to complete the initial preliminary report. All other SAEs will be reported within a period of maximum 15 days after the sponsor has first knowledge of the serious adverse events.

**8.2.3 Suspected unexpected serious adverse reactions (SUSARs)**

Conversion of the OLGB to a RYGB due to untreatable biliary reflux is expected to occur in 5-10% of patients. They will be reported through the web portal *ToetsingOnline*

## Follow-up of adverse events

All AEs will be followed until they have abated, or until a stable situation has been reached. Depending on the event, follow up may require additional tests or medical procedures as indicated, and/or referral to the general physician or a medical specialist.

SAEs need to be reported till end of study within the Netherlands, as defined in the protocol

## [Data Safety Monitoring Board (DSMB) / Safety Committee]

Based on the current experience of the surgeons with the procedures a DSMB is not

considered necessary

# STATISTICAL ANALYSIS

## Populations for analysis

## All efficacy analyses will be conducted using the full analysis set. The full analysis set will include all randomized patients with at least one post-baseline measurement and fulfilling all inclusion criteria, in accordance with the intention to treat principle (ITT).

## The Per-Protocol analysis set is defined as those patients of the full analysis set who completed the study at 60 months and are fully compliant to all exclusion criteria. The Per-protocol analysis set will be used only if the percentage of excluded patients is substantial, i.e. > 15% of the full analysis set.

## The safety analysis set consists of all randomized patients.

## All patients will be analyzed according to allocated treatment strategy, irrespective of treatment strategy actually used. For safety endpoints, analysis will be performed using the treatment strategy that was actually used. An additional “Per-Protocol analysis” will be conducted only if the Per-Protocol analysis set is defined.

## Handling of missing data

## Missing data will remain as missing, and no attempt will be made to estimate or replace missing values. Only observed values will be used in data analyses and presentations.

## Patient demographics and other baseline characteristics

## A schematic summary of the number of eligible patients enrolled and randomized will be presented by treatment strategy group. The number of subjects withdrawn from the study after randomization will be tabulated by treatment strategy group. The time and reason for withdrawal will be listed by treatment strategy group. Potential biases due to withdrawal of subjects will be investigated.

## A complete baseline description of the patients' demographics and disease characteristics will be tabulated by treatment strategy group for the full analysis set. This includes demographics (e.g. age, gender, race) and a detailed baseline description including general medical history, disease specific medical history (including risk factors), physical examination (e.g. weight, height), vital signs and relevant concomitant medication. The baseline values of study endpoints, where applicable, will be included in the presentation of the baseline description.

## Statistical differences will not be formally evaluated. If differences are considered clinically relevant, caution will be taken when interpreting the results of the analyses between groups and the statistical methods may be modified to adjust for this difference. (By definition, differences between the randomised treatment groups at baseline are due to chance only.)

## For the quantitative parameters, the descriptive statistics will be mean with standard deviation or median with range of minimum and maximum, and number of valid observations. Mean and median will be reported to a precision of one decimal place more than the individual measurements; standard deviation will be reported to a precision of two decimal places more than the individual measurements; and range will have the same precision as the individual measurements.

## For qualitative parameters (categorical or ordered), frequency counts and percentages of each category will be calculated by treatment group. Percentages will be reported up to 1 decimal place.

## Primary study parameter(s)

The primary null hypothesis for this study is that there is no significant difference in the mean change in the percent of total weight loss (%TWL) at five years, between patients treated with a TSBL adjusted BP limb length (active group) versus patients treated with a standard BP limb length of 150 cm (control group).

In addition to the descriptive statistics (see the above section : Patient demographics other baseline characteristics), the two treatment strategy groups will be compared using two-sample T-test if parameter is normally distributed. Visual inspection of the distribution, as well as the Shapiro-Wilk test for normality, will used to assess normality. Unless equality of variance between groups is established, the Satterthwaite method for t-statistics and 95% confidence intervals will be used. If data is skewedly distributed, a Mann-Whitney U-test (Wilcoxon two-sample test) will be applied. For the difference in medians, the Hodges-Lehmann estimate will be used with distribution-free 95% confidence intervals.

In addition to the above mentioned analyses, the homogeneity of treatment effect over different strata of patients will be evaluated. In this, differences in treatment effect over separate strata of potentially relevant factors (e.g. gender, baseline BMI, co-morbidities, compliance, etc) will be evaluated. A multivariable model will be used to formally establish homogeneity, i.e. the absence of effect modification for the potentially relevant factors, separately.

## Secondary study parameter(s)

All secondary endpoint parameters (quantitative and qualitative) will be presented as described in section : Patient demographics other baseline characteristics.

For the evaluation of differences between groups, the primary analysis approach will be applied for the quantitative secondairy endpoint parameters..

For the qualitative parameters a Fisher exact test or a Chi-square test will be used.

For the parameters related to the occurrence of side effects, the evaluation focusses on the “absence” of differences. For these analysis estimates of risk differences with 90% confidence intervals, based on the Farrington-Manning Method will be used (non-inferiority setting, one sided approach). Due to sample size constraints, formal hypothesis tests on non-inferiority will not be performed. Multivariable logistic (or log-binomial) regression modelling is not foreseen.

## Annual safety report

An annual safety report will be made by the investigator and provided to the sponsor.

## Other study parameters

**Safety analysis**

All serious adverse events will be summarized and differences between study groups assessed using the same approach as specified for the quantitative secondary endpoint parameters. Relationship to study intervention, i.e. to the measurement of total small bowel length or adjusting the BP-limb length, will be presented. Furthermore, a differentiation between surgical complications and non-surgical serious complications (e.g. excessive weight loss, diarrhea or nutrient deficiencies) will be provided.

Of all non-serious adverse events only surgical complications will be presented. In this, early (within one month) and late (after one month) surgical complications will be presented separately.

All non-serious, non-surgical complications (e.g. excessive weight loss, diarrhea or nutrient deficiencies) are taken into account as secondary efficacy endpoints.

## Interim analysis (if applicable)

The first interim analysis will be performed after 50 patients have been included in order to see whether the number of patients with a TSBL of < 500cm, allocated to the intervention, will not jeopardize the study power.

Interim analyses will be performed after all patients have completed the visits at 1,2,3 and 4 years. The deblinding of the allocated treatment will be performed by an independent person, who is not part of the investigation team

# ETHICAL CONSIDERATIONS

## Regulation statement

The study will be conducted according to the principles of the Declaration of Helsinki Adopted by the 18th WMA General Assembly, Helsinki, Finland, June 1964, and amended by the WMA General Assembly, Seoul, Korea, October 2008 and in accordance with the Medical Research Involving Human Subjects Act (WMO)

## Recruitment and consent

Patients will be asked to participate during their visit to their surgeon before the surgery after they have decided for an OLGB. After short verbal explanation of the study, they will be provided with the written patient information and consent form. After 1-2 weeks they will be approached by telephone. When they have decided to participate they will have a meeting with the investigator on admission the day before surgery. Any questions still not answered will be addressed and the informed consent form will be signed by the patient and the investigator twice (one copy for the patient). After this procedure the patient will be asked to swallow 1 Fitforme WLS primo capsule in order to see whether they arte able to swallow the capsule. If not, the patient will drop out of the study and will be replaced

## Compensation for injury

The sponsor has an insurance which is in accordance with the legal requirements in the Netherlands (Article 7 WMO). This insurance provides cover for damage to research subjects through injury or death caused by the study.

The insurance applies to the damage that becomes apparent during the study or within 4 years after the end of the study.

## Incentives (if applicable)

During the five years of study patients will be provided with daily multivitamin FFM WSL primo for free.

# ADMINISTRATIVE ASPECTS, MONITORING AND PUBLICATION

## Handling and storage of data and documents

All data will be stored in a data file in which the patients are made anonymous by allocating a study number. The log file on names and numbers will be stored separately by the principle investigator.

## Monitoring and Quality Assurance

An independent physician with experience in trials will be asked to monitor the quality of data acquisition and documentation

## Amendments

Amendments are changes made to the research after a favourable opinion by the accredited METC has been given. All amendments will be notified to the METC that gave a favourable opinion.

A ‘substantial amendment’ is defined as an amendment to the terms of the METC application, or to the protocol or any other supporting documentation, that is likely to affect to a significant degree:

- the safety or physical or mental integrity of the subjects of the trial;
- the scientific value of the trial;
- the conduct or management of the trial; or
- the quality or safety of any intervention used in the trial.

All substantial amendments will be notified to the METC and to the competent authority.

Non-substantial amendments will not be notified to the accredited METC and the competent authority, but will be recorded and filed by the sponsor.

## Annual progress report

The sponsor/investigator will submit a summary of the progress of the trial to the accredited METC once a year. Information will be provided on the date of inclusion of the first subject, numbers of subjects included and numbers of subjects that have completed the trial, serious adverse events/ serious adverse reactions, other problems, and amendments.

## Temporary halt and (prematurely) end of study report

The investigator/sponsor will notify the accredited METC of the end of the study within a period of 8 weeks. The end of the study is defined as the last patient’s last visit.
The sponsor will notify the METC immediately of a temporary halt of the study, including the reason of such an action.

In case the study is ended prematurely, the sponsor will notify the accredited METC within 15 days, including the reasons for the premature termination.
 Within one year after the end of the study, the investigator/sponsor will submit a final study report with the results of the study, including any publications/abstracts of the study, to the accredited METC.

## Public disclosure and publication policy

The study is registred in the Dutch Trial Registry (Nederlands Trial Register) : NL 7945.

The results of the study will be published in a peer-reviewed medical journal after agreement of all participating authors. Fitforme will not be involved in the decision to publish nor in content of the of the publication. The CCMO statement concerning publication policy will be followed

# STRUCTURED RISK ANALYSIS

## Potential issues of concern

1. a full measurement of the small bowel length during surgery includes a small risk of laceration of the bowel. The surgeons are experienced in this technique of measuring as they need to measure the distance from Treitz to the planned anastomosis during the standard procedure of the OLGB.

2. the adjusted lengths of the biliopancreatic limb based on total small bowel length are within the currently used lengths in literature and are considered safe. Whether this leads to less or more adverse events in the patients after the surgery is part of the investigation. However, all expected side effects are treatable.

# REFERENCES

1. Charalampos T, Maria N, Vrakopoulou VGZ, Tania T, Raptis D, George

Z, Emmanouil L, Konstantinos A. Tailored One Anastomosis Gastric Bypass: 3-Year Outcomes of 94 Patients.

Obes Surg. 2019 Feb;29(2):542-551. doi: 10.1007/s11695-018-3572-6.

2. Ahuja A, Tantia O, Goyal G, Chaudhuri T, Khanna S, Poddar A,

Gupta S, Majumdar K. MGB-OAGB: Effect of Biliopancreatic Limb Length on Nutritional Deficiency, Weight Loss, and Comorbidity Resolution.

Obes Surg. 2018 Nov;28(11):3439-3445. doi: 10.1007/s11695-018-3405-7.

3. Nergaard BJ, Leifsson BG, Hedenbro J, Gislason H. Gastric bypass with long alimentary limb or long pancreato-biliary limb--long-term results on weight loss, resolution of co-morbidities and metabolic parameters.

Obes Surg. 2014 Oct;24(10):1595-602. doi: 10.1007/s11695-014-1245-7.

4. Zorrilla-Nunez LF, Campbell A, Giambartolomei G, Lo Menzo E, Szomstein S, Rosenthal RJ. The importance of the biliopancreatic limb length in gastric bypass: A systematic review.

Surg Obes Relat Dis. 2019 Jan;15(1):43-49. doi: 10.1016/j.soard.2018.10.013.

5. Tacchino RM. Bowel length: measurement, predictors, and impact on bariatric and metabolic surgery.

Surg Obes Relat Dis. 2015 Mar-Apr;11(2):328-34. doi: 10.1016/j.soard.2014.09.016.

6. Prager G, personal communication

7. Emous M, Wolffenbuttel BHR, Totté E, van Beek AP. The short- to mid-term symptom prevalence of dumping syndrome after primary gastric-bypass surgery and its impact on health-related quality of life.

Surg Obes Relat Dis. 2017 Sep;13(9):1489-1500. doi: 10.1016/j.soard.2017.04.028.
