# Supplementary material for: Effect of tailoring biliopancreatic limb length based on total small bowel length versus standard limb length in one anastomosis gastric bypass: 1-year outcomes of the TAILOR randomized clinical superiority trial
Source: Br J Surg. 2024 Aug 30;111(9):znae219. doi: 10.1093/bjs/znae219 (PMC11363871; doi:10.1093/bjs/znae219)
Supplement: znae219_Supplementary_Data [file znae219_supplementary_data.zip › Supplementary table 1.docx]

| **Table 1.** Reference values used to define nutritional deficiencies | |
| --- | --- |
| **Reference values** | |
| Hemoglobin |  |
| Female | 7.5 – 10.0 mmol/L |
| Male | 8.5 – 11.0 mmol/L |
| Ferritin | Definition of deficiency: < 30 µg/L or  30-50 µg/L and a decrease of over 50 µg/L compared to the prior measurement |
| Potassium | 3.5 – 5.0 mmol/L |
| Magnesium | 0.7 – 1.0 mmol/L |
| Phosphate | 0.8 – 1.4 mmol/L |
| Albumin | 35 - 50 g/L |
| Calcium | 2.2 – 2.6 mmol/L |
| Vitamin A | Definition of deficiency: <0.8 µmol/L |
| Vitamin B1 | 100 - 190 nmol/L |
| Vitamin B6 | 50 - 180 nmol/L |
| Vitamin B12 | Definition of deficiency: <250 pmol/L |
| Vitamin D | 50 - 250 nmol/L |
| Folic acid | 7.3 - 38.5 nmol/L |
| Zinc |  |
| Female | 8.9 - 17.1 umol/L |
| Male | 9.5 – 19.1 umol/L |

## Supplementary table
