# Supplementary material for: Effect of tailoring biliopancreatic limb length based on total small bowel length versus standard limb length in one anastomosis gastric bypass: 1-year outcomes of the TAILOR randomized clinical superiority trial
Source: Br J Surg. 2024 Aug 30;111(9):znae219. doi: 10.1093/bjs/znae219 (PMC11363871; doi:10.1093/bjs/znae219)
Supplement: znae219_Supplementary_Data [file znae219_supplementary_data.zip › Supplementary_Material.docx]

Effect of tailoring biliopancreatic limb length based on total small bowel length vs. standard limb length in one anastomosis gastric bypass: 1-year outcomes of TAILOR randomized clinical superiority trial

Nienke Slagter, M.D. Ph.D. 1,2,3,4, Lindsy van der Laan, M.D. 1,2,3,4, Loek J.M. de Heide, M.D. 1,2, Ewoud H. Jutte M.D. 1,2, Mirjam A. Kaijser, M.D. 1,2, Stefan L. Damen, M.D. 1,2, André P. van Beek, M.D. Ph.D. 3,4, Marloes Emous, M.D. Ph.D.1,2

### Affiliations:

1. Medical Center Leeuwarden, Center for Obesity Northern-Netherlands (CON), Department of Bariatric and Metabolic Surgery, Leeuwarden, the Netherlands
2. Medical Center Leeuwarden, Department of Surgery, Leeuwarden, The Netherlands
3. University of Groningen, University Medical Center Groningen, Post Graduate School of Medicine, Groningen, The Netherlands
4. University of Groningen, University Medical Center Groningen, Department of Endocrinology, Groningen, The Netherlands

**Corresponding author:**

N. Slagter

Email: [N.Slagter@umcg.nl](mailto:N.Slagter@umcg.nl); [nienke.slagter@mcl.nl](mailto:nienke.slagter@mcl.nl)

Center for Obesity Northern Netherlands

Medical Center Leeuwarden

Henri Dunantweg 2

8934 AD Leeuwarden

The Netherlands

Phone: +31 58 286 6969

**Supplementary Materials - Index**

| **Supplementary Methods** |  |
| --- | --- |
| **Supplementary Results** |  |
|  |  |
|  |  |
| **Supplementary Appendixes** |  |
|  |  |
|  |  |
| **Supplementary Figures and Tables** |  |
| \| **Table 1.** Reference values used to define nutritional deficiencies \| \| \| --- \| --- \| \| **Reference values** \| \| \| Hemoglobin \|  \| \| Female \| 7.5 – 10.0 mmol/L \| \| Male \| 8.5 – 11.0 mmol/L \| \| Ferritin \| Definition of deficiency: < 30 µg/L or  30-50 µg/L and a decrease of over 50 µg/L compared to the prior measurement \| \| Potassium \| 3.5 – 5.0 mmol/L \| \| Magnesium \| 0.7 – 1.0 mmol/L \| \| Phosphate \| 0.8 – 1.4 mmol/L \| \| Albumin \| 35 - 50 g/L \| \| Calcium \| 2.2 – 2.6 mmol/L \| \| Vitamin A \| Definition of deficiency: <0.8 µmol/L \| \| Vitamin B1 \| 100 - 190 nmol/L \| \| Vitamin B6 \| 50 - 180 nmol/L \| \| Vitamin B12 \| Definition of deficiency: <250 pmol/L \| \| Vitamin D \| 50 - 250 nmol/L \| \| Folic acid \| 7.3 - 38.5 nmol/L \| \| Zinc \|  \| \| Female \| 8.9 - 17.1 umol/L \| \| Male \| 9.5 – 19.1 umol/L \|   **Figure 1.** Remission of comorbidities at one-year follow-up  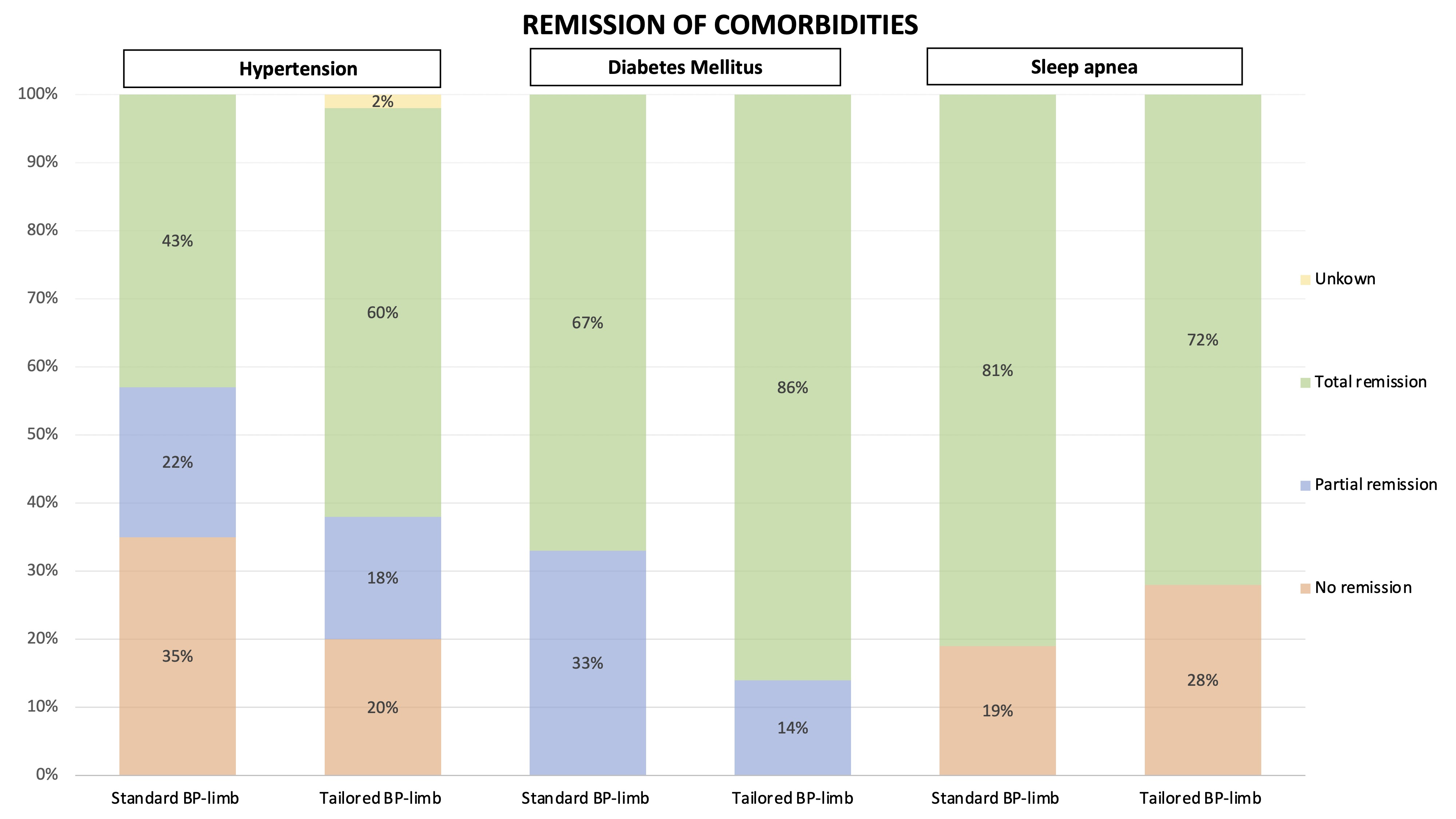 |  |

**Supplementary Methods**

Supplementary file 1 – Study protocol

**Supplementary Results**

**Supplementary Appendixes**

**Supplementary Figures and Tables**

**References**
