# Supplementary figures and images for: Effect of tailoring biliopancreatic limb length based on total small bowel length versus standard limb length in one anastomosis gastric bypass: 1-year outcomes of the TAILOR randomized clinical superiority trial
Source: Br J Surg. 2024 Aug 30;111(9):znae219. doi: 10.1093/bjs/znae219 (PMC11363871; doi:10.1093/bjs/znae219)

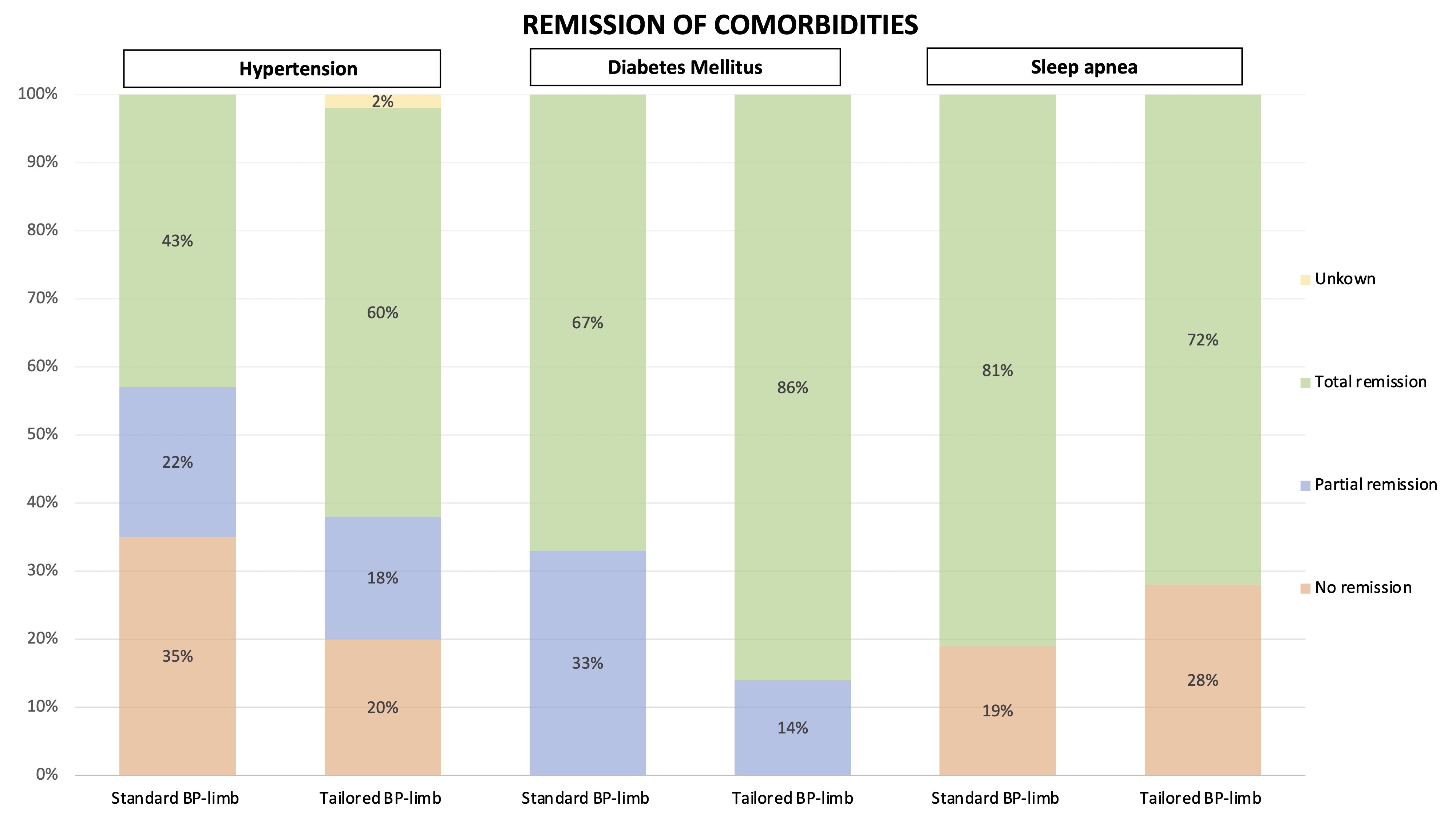

Supplement: znae219_Supplementary_Data [file znae219_supplementary_data.zip › Supplementary fig 1- Remission of comos def.jpg]
